# Supplementary material for: Key drivers of fertility levels and differentials in India, at the national, state and population subgroup levels, 2015–2016: An application of Bongaarts’ proximate determinants model
Source: PLoS One. 2022 Feb 7;17(2):e0263532. doi: 10.1371/journal.pone.0263532 (PMC8820640; doi:10.1371/journal.pone.0263532)
Supplement: S1 Table — (DOCX) [file pone.0263532.s001.docx]

**S1 Table: Selected indicators of sexual and reproductive behaviors and unweighted sample size according to social and economic subgroup: National and by state, India 2015-16**

|  |  |  | Among married women | | | | | | |  |
| --- | --- | --- | --- | --- | --- | --- | --- | --- | --- | --- |
| **Sub-group** |  | **Among all women, % currently married*** | **% using modern methods** | **% using tradition-**  **al methods** | **% with unmet need for modern methods** | **Average contra-**  **ceptive use effective-**  **ness** | **Average months of post-**  **partum infecund-**  **ability** | **Abortion**  **rate** | **% had non-contra-**  **ceptive**  **hyster-**  **ectomy** | **Number of Women: Unweighted Ns** |
| **India** |  | 73% | 48% | 6% | 19% | 96% | 7 | 47.0 | 4% | 699,686 |
| Residence |  |  |  |  |  |  |  |  |  |  |
|  | Urban | 71% | 51% | 6% | 18% | 96% | 5 | 45.0 | 3% | 204,735 |
|  | Rural | 74% | 46% | 6% | 19% | 96% | 7 | 49.0 | 4% | 494,951 |
| Education |  |  |  |  |  |  |  |  |  |  |
|  | <5 years | 87% | 50% | 5% | 16% | 97% | 8 | 38.8 | 6% | 237,494 |
|  | 5-9 years | 73% | 49% | 6% | 19% | 96% | 7 | 46.1 | 3% | 225,316 |
|  | 10 years plus | 60% | 43% | 6% | 22% | 95% | 5 | 56.1 | 2% | 236,876 |
| Wealth status |  |  |  |  |  |  |  |  |  |  |
|  | Low | 74% | 40% | 6% | 21% | 96% | 8 | 55.3 | 5% | 252,734 |
|  | Middle | 73% | 51% | 6% | 17% | 97% | 6 | 42.5 | 4% | 235,682 |
|  | High | 72% | 52% | 6% | 18% | 96% | 4 | 43.3 | 3% | 211,270 |
| Caste |  |  |  |  |  |  |  |  |  |  |
|  | Scheduled caste/tribe | 73% | 48% | 5% | 18% | 97% | 8 | 45.8 | 4% | 251,946 |
|  | Other Backward Classes | 73% | 47% | 5% | 18% | 97% | 6 | 49.6 | 4% | 273,700 |
|  | Others | 73% | 50% | 8% | 20% | 95% | 6 | 45.7 | 4% | 170,730 |
| **North** |  |  |  |  |  |  |  |  |  |  |
| **Haryana** | | 75% | 59% | 4% | 14% | 96% | 6 | 51.3 | 3% | 21,654 |
| Residence |  |  |  |  |  |  |  |  |  |  |
|  | Urban | 75% | 55% | 5% | 16% | 96% | 6 | 58.1 | 3% | 7,562 |
|  | Rural | 75% | 62% | 4% | 12% | 97% | 6 | 44.5 | 2% | 14,092 |
| Education |  |  |  |  |  |  |  |  |  |  |
|  | <5 years | 90% | 60% | 3% | 13% | 98% | 7 | 52.2 | 4% | 5,478 |
|  | 5-9 years | 77% | 62% | 5% | 13% | 97% | 7 | 45.1 | 2% | 6,121 |
|  | 10 years plus | 66% | 57% | 5% | 15% | 95% | 5 | 56.6 | 2% | 10,055 |
| Wealth status |  |  |  |  |  |  |  |  |  |  |
|  | Low | 73% | 42% | 3% | 23% | 97% | 6 | 79.9 | 2% | 1,368 |
|  |  | **Among all women, % currently married*** | **% using modern methods** | **% using tradition-**  **al methods** | **% with unmet need for modern methods** | **Average contra-**  **ceptive use effective-**  **ness** | **Average months of post-**  **partum infecund-**  **ability** | **Abortion**  **rate** | **% had non-contra-**  **ceptive**  **hyster-**  **ectomy** | **Number of Women: Unweighted Ns** |
|  | Middle | 74% | 57% | 4% | 14% | 97% | 7 | 41.6 | 3% | 5,892 |
|  | High | 76% | 62% | 5% | 13% | 96% | 5 | 32.3 | 3% | 14,394 |
| Caste |  |  |  |  |  |  |  |  |  |  |
|  | Scheduled caste/tribe | 74% | 61% | 4% | 13% | 97% | 7 | 50.5 | 1% | 6,001 |
|  | Other Backward Classes | 76% | 60% | 5% | 14% | 96% | 6 | 51.4 | 3% | 9,862 |
|  | Others | 75% | 58% | 4% | 13% | 96% | 5 | 51.9 | 4% | 5,740 |
|  |  |  |  |  |  |  |  |  |  |  |
| **Himachal Pradesh** | | 75% | 52% | 5% | 21% | 96% | 3 | 51.3 | 3% | 9,929 |
| Residence |  |  |  |  |  |  |  |  |  |  |
|  | Urban | 71% | 50% | 7% | 25% | 95% | 0 | 55.4 | 3% | 692 |
|  | Rural | 76% | 52% | 5% | 20% | 96% | 3 | 47.2 | 3% | 9,237 |
| Education |  |  |  |  |  |  |  |  |  |  |
|  | <5 years | 90% | 67% | 2% | 9% | 99% | 4 | 28.0 | 4% | 1,509 |
|  | 5-9 years | 84% | 57% | 3% | 14% | 97% | 3 | 42.1 | 4% | 2,810 |
|  | 10 years plus | 68% | 45% | 6% | 28% | 94% | 3 | 83.8 | 2% | 5,610 |
| Wealth status |  |  |  |  |  |  |  |  |  |  |
|  | Low | 73% | 58% | 5% | 16% | 97% | 1 | 41.7 | 1% | 855 |
|  | Middle | 74% | 56% | 4% | 18% | 97% | 3 | 49.6 | 2% | 4,233 |
|  | High | 76% | 48% | 6% | 23% | 95% | 3 | 62.5 | 3% | 4,841 |
| Caste |  |  |  |  |  |  |  |  |  |  |
|  | Scheduled caste/tribe | 74% | 55% | 4% | 18% | 97% | 3 | 46.8 | 3% | 3,678 |
|  | Other Backward Classes | 74% | 48% | 5% | 22% | 95% | 6 | 54.7 | 2% | 1,126 |
|  | Others | 76% | 52% | 5% | 21% | 96% | 2 | 52.3 | 2% | 5,063 |
|  |  |  |  |  |  |  |  |  |  |  |
| **Jammu & Kashmir** | | 63% | 46% | 11% | 24% | 94% | 7 | 51.3 | 4% | 23,800 |
| Residence |  |  |  |  |  |  |  |  |  |  |
|  | Urban | 61% | 56% | 9% | 18% | 95% | 4 | 40.1 | 3% | 4,325 |
|  | Rural | 64% | 42% | 12% | 26% | 93% | 8 | 62.5 | 4% | 19,475 |
| Education |  |  |  |  |  |  |  |  |  |  |
|  | <5 years | 88% | 49% | 10% | 21% | 95% | 8 | 42.4 | 5% | 8,217 |
|  | 5-9 years | 58% | 44% | 13% | 27% | 93% | 7 | 55.8 | 3% | 7,350 |
|  |  | **Among all women, % currently married*** | **% using modern methods** | **% using tradition-**  **al methods** | **% with unmet need for modern methods** | **Average contra-**  **ceptive use effective-**  **ness** | **Average months of post-**  **partum infecund-**  **ability** | **Abortion**  **rate** | **% had non-contra-**  **ceptive**  **hyster-**  **ectomy** | **Number of Women: Unweighted Ns** |
|  | 10 years plus | 47% | 44% | 10% | 24% | 93% | 5 | 55.6 | 2% | 8,233 |
| Wealth status |  |  |  |  |  |  |  |  |  |  |
|  | Low | 66% | 40% | 11% | 26% | 94% | 8 | 62.8 | 3% | 6,405 |
|  | Middle | 60% | 46% | 12% | 24% | 94% | 7 | 45.7 | 4% | 10,026 |
|  | High | 64% | 49% | 11% | 22% | 94% | 6 | 45.4 | 4% | 7,369 |
| Caste |  |  |  |  |  |  |  |  |  |  |
|  | Scheduled caste/tribe | 69% | 45% | 7% | 23% | 95% | 7 | 64.2 | 3% | 5,489 |
|  | Other Backward Classes | 63% | 49% | 7% | 19% | 95% | 4 | 45.4 | 3% | 1,136 |
|  | Others | 61% | 46% | 13% | 24% | 94% | 7 | 44.3 | 4% | 17,126 |
|  |  |  |  |  |  |  |  |  |  |  |
| **Punjab** |  | 71% | 66% | 9% | 16% | 95% | 4 | 51.3 | 2% | 19,484 |
| Residence |  |  |  |  |  |  |  |  |  |  |
|  | Urban | 72% | 65% | 11% | 17% | 94% | 4 | 45.6 | 2% | 7,148 |
|  | Rural | 70% | 67% | 8% | 15% | 96% | 4 | 57.0 | 2% | 12,336 |
| Education |  |  |  |  |  |  |  |  |  |  |
|  | <5 years | 89% | 75% | 6% | 10% | 97% | 6 | 34.6 | 3% | 3,657 |
|  | 5-9 years | 75% | 67% | 9% | 16% | 95% | 4 | 54.9 | 3% | 5,378 |
|  | 10 years plus | 63% | 62% | 11% | 18% | 94% | 3 | 64.4 | 2% | 10,449 |
| Wealth status |  |  |  |  |  |  |  |  |  |  |
|  | Low | 71% | 68% | 5% | 12% | 97% | 4 | 55.0 | 1% | 494 |
|  | Middle | 69% | 69% | 8% | 14% | 96% | 6 | 47.2 | 2% | 3,957 |
|  | High | 71% | 66% | 10% | 16% | 95% | 3 | 51.7 | 3% | 15,033 |
| Caste |  |  |  |  |  |  |  |  |  |  |
|  | Scheduled caste/tribe | 68% | 68% | 8% | 14% | 96% | 4 | 54.1 | 2% | 7,777 |
|  | Other Backward Classes | 72% | 65% | 10% | 16% | 95% | 4 | 44.9 | 2% | 3,645 |
|  | Others | 72% | 66% | 11% | 17% | 95% | 4 | 54.9 | 3% | 8,058 |
|  |  |  |  |  |  |  |  |  |  |  |
| **Rajasthan** |  | 75% | 54% | 6% | 19% | 96% | 4 | 51.3 | 2% | 41,965 |
| Residence |  |  |  |  |  |  |  |  |  |  |
|  | Urban | 71% | 58% | 6% | 19% | 95% | 3 | 51.5 | 2% | 11,428 |
|  | Rural | 76% | 52% | 6% | 18% | 97% | 4 | 51.0 | 2% | 30,537 |
|  |  | **Among all women, % currently married*** | **% using modern methods** | **% using tradition-**  **al methods** | **% with unmet need for modern methods** | **Average contra-**  **ceptive use effective-**  **ness** | **Average months of post-**  **partum infecund-**  **ability** | **Abortion**  **rate** | **% had non-contra-**  **ceptive**  **hyster-**  **ectomy** | **Number of Women: Unweighted Ns** |
| Education |  |  |  |  |  |  |  |  |  |  |
|  | <5 years | 89% | 58% | 6% | 15% | 97% | 5 | 36.9 | 2% | 18,802 |
|  | 5-9 years | 70% | 49% | 7% | 22% | 96% | 4 | 57.6 | 1% | 12,848 |
|  | 10 years plus | 54% | 47% | 7% | 22% | 94% | 3 | 59.4 | 1% | 10,315 |
| Wealth status |  |  |  |  |  |  |  |  |  |  |
|  | Low | 76% | 48% | 7% | 19% | 97% | 5 | 53.2 | 2% | 13,758 |
|  | Middle | 74% | 54% | 6% | 18% | 97% | 4 | 51.7 | 2% | 14,220 |
|  | High | 74% | 58% | 6% | 18% | 96% | 3 | 48.9 | 2% | 13,987 |
| Caste |  |  |  |  |  |  |  |  |  |  |
|  | Scheduled caste/tribe | 74% | 51% | 6% | 19% | 97% | 5 | 52.3 | 2% | 13,667 |
|  | Other Backward Classes | 76% | 54% | 6% | 18% | 96% | 4 | 51.7 | 2% | 19,786 |
|  | Others | 72% | 58% | 6% | 18% | 96% | 4 | 49.9 | 2% | 8,189 |
|  |  |  |  |  |  |  |  |  |  |  |
| **Uttarakhand** |  | 68% | 49% | 4% | 20% | 95% | 6 | 51.3 | 3% | 17,300 |
| Residence |  |  |  |  |  |  |  |  |  |  |
|  | Urban | 67% | 48% | 5% | 19% | 94% | 4 | 47.2 | 3% | 5,213 |
|  | Rural | 69% | 50% | 3% | 20% | 96% | 8 | 55.4 | 3% | 12,087 |
| Education |  |  |  |  |  |  |  |  |  |  |
|  | <5 years | 87% | 49% | 4% | 18% | 96% | 7 | 47.0 | 4% | 4,017 |
|  | 5-9 years | 69% | 52% | 3% | 18% | 96% | 6 | 49.1 | 3% | 5,551 |
|  | 10 years plus | 57% | 47% | 5% | 22% | 94% | 6 | 57.7 | 2% | 7,732 |
| Wealth status |  |  |  |  |  |  |  |  |  |  |
|  | Low | 68% | 50% | 3% | 19% | 97% | 7 | 53.4 | 2% | 3,150 |
|  | Middle | 68% | 48% | 4% | 20% | 96% | 8 | 54.2 | 2% | 7,283 |
|  | High | 68% | 50% | 5% | 19% | 94% | 4 | 46.3 | 3% | 6,867 |
| Caste |  |  |  |  |  |  |  |  |  |  |
|  | Scheduled caste/tribe | 66% | 48% | 4% | 18% | 96% | 8 | 48.2 | 3% | 4,445 |
|  | Other Backward Classes | 67% | 43% | 5% | 20% | 94% | 3 | 52.2 | 3% | 3,804 |
|  | Others | 70% | 54% | 4% | 20% | 96% | 8 | 53.4 | 2% | 8,932 |
| **Central** |  |  |  |  |  |  |  |  |  |  |
| **Chhattisgarh** |  | 69% | 55% | 3% | 14% | 98% | 6 | 56.6 | 1% | 25,172 |
|  |  | **Among all women, % currently married*** | **% using modern methods** | **% using tradition-**  **al methods** | **% with unmet need for modern methods** | **Average contra-**  **ceptive use effective-**  **ness** | **Average months of post-**  **partum infecund-**  **ability** | **Abortion**  **rate** | **% had non-contra-**  **ceptive**  **hyster-**  **ectomy** | **Number of Women: Unweighted Ns** |
| Residence |  |  |  |  |  |  |  |  |  |  |
|  | Urban | 67% | 57% | 4% | 15% | 97% | 4 | 56.1 | 1% | 7,015 |
|  | Rural | 70% | 54% | 3% | 14% | 98% | 6 | 57.1 | 1% | 18,157 |
| Education |  |  |  |  |  |  |  |  |  |  |
|  | <5 years | 86% | 58% | 2% | 11% | 99% | 7 | 40.8 | 2% | 9,150 |
|  | 5-9 years | 66% | 55% | 3% | 15% | 98% | 6 | 55.5 | 1% | 9,398 |
|  | 10 years plus | 52% | 45% | 5% | 21% | 96% | 4 | 73.5 | 1% | 6,624 |
| Wealth status |  |  |  |  |  |  |  |  |  |  |
|  | Low | 69% | 50% | 3% | 14% | 98% | 7 | 58.5 | 1% | 13,127 |
|  | Middle | 69% | 59% | 3% | 14% | 98% | 5 | 52.6 | 1% | 6,323 |
|  | High | 70% | 59% | 4% | 16% | 97% | 4 | 58.8 | 2% | 5,722 |
| Caste |  |  |  |  |  |  |  |  |  |  |
|  | Scheduled caste/tribe | 69% | 48% | 3% | 15% | 98% | 6 | 61.2 | 1% | 12,257 |
|  | Other Backward Classes | 69% | 60% | 3% | 13% | 98% | 6 | 49.2 | 2% | 10,786 |
|  | Others | 71% | 54% | 6% | 18% | 96% | 5 | 59.4 | 1% | 2,110 |
|  |  |  |  |  |  |  |  |  |  |  |
| **Madhya Pradesh** | | 75% | 50% | 2% | 14% | 98% | 7 | 57.3 | 6% | 62,803 |
| Residence |  |  |  |  |  |  |  |  |  |  |
|  | Urban | 70% | 49% | 3% | 16% | 97% | 5 | 61.5 | 6% | 18,762 |
|  | Rural | 76% | 50% | 1% | 13% | 98% | 8 | 53.0 | 7% | 44,041 |
| Education |  |  |  |  |  |  |  |  |  |  |
|  | <5 years | 91% | 56% | 1% | 9% | 99% | 8 | 33.0 | 8% | 25,917 |
|  | 5-9 years | 68% | 44% | 2% | 17% | 98% | 7 | 61.6 | 5% | 23,048 |
|  | 10 years plus | 57% | 41% | 3% | 22% | 95% | 5 | 77.2 | 4% | 13,838 |
| Wealth status |  |  |  |  |  |  |  |  |  |  |
|  | Low | 77% | 48% | 1% | 13% | 99% | 8 | 53.9 | 6% | 29,307 |
|  | Middle | 74% | 51% | 2% | 14% | 98% | 6 | 56.1 | 7% | 17,029 |
|  | High | 72% | 50% | 3% | 16% | 97% | 5 | 61.8 | 7% | 16,467 |
| Caste |  |  |  |  |  |  |  |  |  |  |
|  | Scheduled caste/tribe | 74% | 49% | 1% | 13% | 99% | 7 | 54.0 | 6% | 23,642 |
|  | Other Backward Classes | 75% | 51% | 2% | 14% | 98% | 7 | 55.1 | 6% | 28,247 |
|  |  | **Among all women, % currently married*** | **% using modern methods** | **% using tradition-**  **al methods** | **% with unmet need for modern methods** | **Average contra-**  **ceptive use effective-**  **ness** | **Average months of post-**  **partum infecund-**  **ability** | **Abortion**  **rate** | **% had non-contra-**  **ceptive**  **hyster-**  **ectomy** | **Number of Women: Unweighted Ns** |
|  | Others | 73% | 48% | 3% | 17% | 97% | 6 | 62.8 | 7% | 10,664 |
|  |  |  |  |  |  |  |  |  |  |  |
| **Uttar Pradesh** |  | 68% | 32% | 14% | 32% | 92% | 6 | 61.1 | 3% | 97,661 |
| Residence |  |  |  |  |  |  |  |  |  |  |
|  | Urban | 64% | 40% | 16% | 29% | 91% | 4 | 49.5 | 3% | 26,586 |
|  | Rural | 69% | 29% | 13% | 33% | 92% | 6 | 72.6 | 3% | 71,075 |
| Education |  |  |  |  |  |  |  |  |  |  |
|  | <5 years | 87% | 31% | 14% | 31% | 92% | 7 | 56.0 | 4% | 37,616 |
|  | 5-9 years | 61% | 32% | 14% | 32% | 92% | 5 | 61.1 | 2% | 28,289 |
|  | 10 years plus | 51% | 32% | 13% | 33% | 91% | 5 | 66.1 | 2% | 31,756 |
| Wealth status |  |  |  |  |  |  |  |  |  |  |
|  | Low | 70% | 26% | 13% | 34% | 92% | 7 | 74.2 | 3% | 42,650 |
|  | Middle | 67% | 32% | 14% | 32% | 92% | 5 | 60.6 | 3% | 28,814 |
|  | High | 66% | 41% | 14% | 28% | 92% | 3 | 48.5 | 3% | 26,197 |
| Caste |  |  |  |  |  |  |  |  |  |  |
|  | Scheduled caste/tribe | 69% | 30% | 13% | 32% | 92% | 7 | 64.6 | 2% | 22,669 |
|  | Other Backward Classes | 68% | 31% | 14% | 33% | 92% | 5 | 62.6 | 3% | 53,653 |
|  | Others | 67% | 36% | 13% | 30% | 92% | 4 | 56.0 | 3% | 21,189 |
| **East** |  |  |  |  |  |  |  |  |  |  |
| **Bihar** |  | 77% | 23% | 1% | 22% | 98% | 9 | 49.4 | 11% | 45,812 |
| Residence |  |  |  |  |  |  |  |  |  |  |
|  | Urban | 71% | 32% | 2% | 22% | 97% | 7 | 46.6 | 8% | 6,096 |
|  | Rural | 78% | 22% | 1% | 22% | 99% | 9 | 52.2 | 12% | 39,716 |
| Education |  |  |  |  |  |  |  |  |  |  |
|  | <5 years | 90% | 23% | 1% | 20% | 99% | 9 | 42.5 | 13% | 23,961 |
|  | 5-9 years | 64% | 23% | 1% | 24% | 98% | 8 | 51.6 | 9% | 11,094 |
|  | 10 years plus | 61% | 23% | 2% | 26% | 97% | 8 | 54.2 | 7% | 10,757 |
| Wealth status |  |  |  |  |  |  |  |  |  |  |
|  | Low | 79% | 21% | 1% | 22% | 99% | 9 | 52.7 | 11% | 31,117 |
|  | Middle | 73% | 27% | 1% | 21% | 98% | 8 | 47.5 | 12% | 10,808 |
|  | High | 72% | 32% | 3% | 23% | 97% | 6 | 48.0 | 11% | 3,887 |
|  |  | **Among all women, % currently married*** | **% using modern methods** | **% using tradition-**  **al methods** | **% with unmet need for modern methods** | **Average contra-**  **ceptive use effective-**  **ness** | **Average months of post-**  **partum infecund-**  **ability** | **Abortion**  **rate** | **% had non-contra-**  **ceptive**  **hyster-**  **ectomy** | **Number of Women: Unweighted Ns** |
| Caste |  |  |  |  |  |  |  |  |  |  |
|  | Scheduled caste/tribe | 80% | 21% | 0% | 22% | 99% | 9 | 49.4 | 11% | 10,217 |
|  | Other Backward Classes | 78% | 24% | 1% | 22% | 98% | 9 | 47.5 | 12% | 27,295 |
|  | Others | 72% | 24% | 1% | 24% | 98% | 8 | 51.3 | 12% | 8,138 |
|  |  |  |  |  |  |  |  |  |  |  |
| **Jharkhand** |  | 75% | 37% | 3% | 21% | 97% | 9 | 52.7 | 5% | 29,046 |
| Residence |  |  |  |  |  |  |  |  |  |  |
|  | Urban | 67% | 43% | 4% | 20% | 97% | 9 | 48.2 | 5% | 7,551 |
|  | Rural | 77% | 36% | 3% | 22% | 98% | 10 | 57.1 | 4% | 21,495 |
| Education |  |  |  |  |  |  |  |  |  |  |
|  | <5 years | 88% | 40% | 2% | 18% | 98% | 10 | 41.9 | 5% | 12,569 |
|  | 5-9 years | 69% | 38% | 3% | 22% | 97% | 10 | 51.9 | 5% | 8,188 |
|  | 10 years plus | 59% | 31% | 5% | 28% | 95% | 8 | 64.2 | 3% | 8,289 |
| Wealth status |  |  |  |  |  |  |  |  |  |  |
|  | Low | 76% | 34% | 2% | 22% | 98% | 10 | 59.1 | 4% | 18,064 |
|  | Middle | 73% | 45% | 4% | 19% | 97% | 8 | 47.6 | 5% | 6,614 |
|  | High | 69% | 43% | 5% | 22% | 96% | 7 | 51.3 | 5% | 4,368 |
| Caste |  |  |  |  |  |  |  |  |  |  |
|  | Scheduled caste/tribe | 73% | 30% | 3% | 23% | 97% | 10 | 58.5 | 4% | 12,107 |
|  | Other Backward Classes | 77% | 42% | 3% | 20% | 98% | 9 | 49.4 | 5% | 13,800 |
|  | Others | 72% | 41% | 4% | 21% | 97% | 7 | 50.1 | 6% | 3,064 |
|  |  |  |  |  |  |  |  |  |  |  |
| **Odisha** |  | 71% | 45% | 12% | 26% | 94% | 14 | 52.7 | 2% | 33,721 |
| Residence |  |  |  |  |  |  |  |  |  |  |
|  | Urban | 70% | 48% | 13% | 26% | 94% | 13 | 52.3 | 2% | 6,646 |
|  | Rural | 72% | 45% | 12% | 25% | 94% | 14 | 53.0 | 3% | 27,075 |
| Education |  |  |  |  |  |  |  |  |  |  |
|  | <5 years | 84% | 49% | 10% | 21% | 95% | 14 | 40.3 | 2% | 12,582 |
|  | 5-9 years | 72% | 45% | 12% | 27% | 94% | 13 | 55.8 | 3% | 12,109 |
|  | 10 years plus | 54% | 39% | 15% | 32% | 92% | 13 | 61.8 | 2% | 9,030 |
| Wealth status |  |  |  |  |  |  |  |  |  |  |
|  |  | **Among all women, % currently married*** | **% using modern methods** | **% using tradition-**  **al methods** | **% with unmet need for modern methods** | **Average contra-**  **ceptive use effective-**  **ness** | **Average months of post-**  **partum infecund-**  **ability** | **Abortion**  **rate** | **% had non-contra-**  **ceptive**  **hyster-**  **ectomy** | **Number of Women: Unweighted Ns** |
|  | Low | 70% | 44% | 11% | 24% | 95% | 13 | 50.7 | 2% | 19,466 |
|  | Middle | 73% | 47% | 13% | 26% | 94% | 15 | 52.6 | 3% | 9,646 |
|  | High | 74% | 47% | 14% | 29% | 93% | 12 | 54.7 | 3% | 4,609 |
| Caste |  |  |  |  |  |  |  |  |  |  |
|  | Scheduled caste/tribe | 71% | 44% | 11% | 24% | 94% | 13 | 49.8 | 2% | 15,787 |
|  | Other Backward Classes | 73% | 47% | 13% | 26% | 94% | 16 | 50.6 | 2% | 11,698 |
|  | Others | 72% | 44% | 12% | 27% | 94% | 13 | 57.6 | 4% | 6,081 |
|  |  |  |  |  |  |  |  |  |  |  |
| **West Bengal** |  | 78% | 57% | 14% | 21% | 94% | 14 | 52.7 | 3% | 17,668 |
| Residence |  |  |  |  |  |  |  |  |  |  |
|  | Urban | 74% | 53% | 16% | 24% | 93% | 10 | 57.4 | 3% | 4,908 |
|  | Rural | 80% | 59% | 13% | 20% | 94% | 15 | 47.9 | 3% | 12,760 |
| Education |  |  |  |  |  |  |  |  |  |  |
|  | <5 years | 87% | 64% | 9% | 15% | 96% | 15 | 44.6 | 4% | 6,606 |
|  | 5-9 years | 79% | 55% | 16% | 23% | 93% | 14 | 53.7 | 2% | 6,658 |
|  | 10 years plus | 66% | 48% | 20% | 29% | 91% | 13 | 59.7 | 2% | 4,404 |
| Wealth status |  |  |  |  |  |  |  |  |  |  |
|  | Low | 79% | 60% | 10% | 18% | 95% | 14 | 54.9 | 3% | 8,695 |
|  | Middle | 79% | 56% | 16% | 23% | 93% | 13 | 48.3 | 3% | 6,134 |
|  | High | 76% | 51% | 19% | 27% | 92% | 15 | 54.8 | 3% | 2,839 |
| Caste |  |  |  |  |  |  |  |  |  |  |
|  | Scheduled caste/tribe | 79% | 61% | 12% | 18% | 95% | 17 | 42.3 | 3% | 6,576 |
|  | Other Backward Classes | 75% | 59% | 13% | 21% | 94% | 12 | 57.8 | 3% | 2,208 |
|  | Others | 78% | 54% | 15% | 24% | 93% | 14 | 57.9 | 3% | 8,598 |
| **Northeast** |  |  |  |  |  |  |  |  |  |  |
| **Arunachal Pradesh** | | 71% | 27% | 5% | 27% | 94% | 11 | 63.6 | 6% | 14,294 |
| Residence |  |  |  |  |  |  |  |  |  |  |
|  | Urban | 64% | 23% | 3% | 28% | 95% | 12 | 70.4 | 7% | 3,123 |
|  | Rural | 74% | 28% | 6% | 26% | 94% | 11 | 56.7 | 5% | 11,171 |
| Education |  |  |  |  |  |  |  |  |  |  |
|  | <5 years | 87% | 27% | 5% | 22% | 95% | 11 | 49.2 | 6% | 5,674 |
|  |  | **Among all women, % currently married*** | **% using modern methods** | **% using tradition-**  **al methods** | **% with unmet need for modern methods** | **Average contra-**  **ceptive use effective-**  **ness** | **Average months of post-**  **partum infecund-**  **ability** | **Abortion**  **rate** | **% had non-contra-**  **ceptive**  **hyster-**  **ectomy** | **Number of Women: Unweighted Ns** |
|  | 5-9 years | 69% | 31% | 6% | 28% | 94% | 11 | 61.4 | 6% | 4,318 |
|  | 10 years plus | 54% | 20% | 4% | 32% | 94% | 11 | 80.2 | 5% | 4,302 |
| Wealth status |  |  |  |  |  |  |  |  |  |  |
|  | Low | 77% | 27% | 7% | 27% | 94% | 10 | 56.9 | 4% | 5,392 |
|  | Middle | 70% | 29% | 4% | 25% | 95% | 11 | 60.5 | 6% | 6,109 |
|  | High | 64% | 22% | 3% | 28% | 95% | 13 | 73.4 | 9% | 2,793 |
| Caste |  |  |  |  |  |  |  |  |  |  |
|  | Scheduled caste/tribe | 70% | 23% | 4% | 26% | 95% | 12 | 74.7 | 6% | 11,902 |
|  | Other Backward Classes | 75% | 37% | 8% | 26% | 94% | 10 | 59.9 | 5% | 654 |
|  | Others | 73% | 39% | 10% | 27% | 93% | 8 | 56.2 | 3% | 1,640 |
|  |  |  |  |  |  |  |  |  |  |  |
| **Assam** |  | 72% | 37% | 15% | 30% | 91% | 11 | 66.2 | 2% | 28,447 |
| Residence |  |  |  |  |  |  |  |  |  |  |
|  | Urban | 67% | 38% | 16% | 29% | 91% | 8 | 62.6 | 3% | 3,811 |
|  | Rural | 73% | 37% | 15% | 30% | 91% | 11 | 69.8 | 2% | 24,636 |
| Education |  |  |  |  |  |  |  |  |  |  |
|  | <5 years | 83% | 38% | 11% | 25% | 93% | 12 | 62.1 | 2% | 9,501 |
|  | 5-9 years | 71% | 38% | 15% | 30% | 91% | 11 | 69.3 | 2% | 11,481 |
|  | 10 years plus | 61% | 32% | 22% | 37% | 89% | 10 | 67.2 | 2% | 7,465 |
| Wealth status |  |  |  |  |  |  |  |  |  |  |
|  | Low | 74% | 38% | 13% | 28% | 92% | 11 | 72.2 | 1% | 15,710 |
|  | Middle | 71% | 37% | 17% | 30% | 91% | 12 | 65.9 | 2% | 9,294 |
|  | High | 67% | 34% | 21% | 33% | 90% | 10 | 60.5 | 3% | 3,443 |
| Caste |  |  |  |  |  |  |  |  |  |  |
|  | Scheduled caste/tribe | 71% | 37% | 17% | 30% | 91% | 12 | 63.8 | 3% | 8,224 |
|  | Other Backward Classes | 69% | 36% | 17% | 30% | 92% | 11 | 64.2 | 3% | 7,682 |
|  | Others | 75% | 38% | 14% | 29% | 91% | 10 | 70.5 | 1% | 12,304 |
|  |  |  |  |  |  |  |  |  |  |  |
| **Manipur** |  | 65% | 13% | 11% | 41% | 90% | 8 | 63.6 | 3% | 13,593 |
| Residence |  |  |  |  |  |  |  |  |  |  |
|  | Urban | 61% | 13% | 12% | 42% | 90% | 12 | 63.1 | 3% | 4,966 |
|  |  | **Among all women, % currently married*** | **% using modern methods** | **% using tradition-**  **al methods** | **% with unmet need for modern methods** | **Average contra-**  **ceptive use effective-**  **ness** | **Average months of post-**  **partum infecund-**  **ability** | **Abortion**  **rate** | **% had non-contra-**  **ceptive**  **hyster-**  **ectomy** | **Number of Women: Unweighted Ns** |
|  | Rural | 67% | 13% | 10% | 40% | 90% | 7 | 64.0 | 2% | 8,627 |
| Education |  |  |  |  |  |  |  |  |  |  |
|  | <5 years | 80% | 14% | 7% | 34% | 92% | 6 | 58.9 | 3% | 2,612 |
|  | 5-9 years | 62% | 14% | 11% | 43% | 90% | 8 | 66.7 | 2% | 5,164 |
|  | 10 years plus | 61% | 11% | 13% | 43% | 89% | 10 | 65.2 | 2% | 5,817 |
| Wealth status |  |  |  |  |  |  |  |  |  |  |
|  | Low | 65% | 12% | 8% | 39% | 91% | 6 | 64.6 | 2% | 4,702 |
|  | Middle | 65% | 13% | 12% | 42% | 90% | 9 | 64.0 | 3% | 6,431 |
|  | High | 64% | 12% | 12% | 42% | 89% | 12 | 62.2 | 3% | 2,460 |
| Caste |  |  |  |  |  |  |  |  |  |  |
|  | Scheduled caste/tribe | 64% | 12% | 8% | 37% | 91% | 6 | 61.3 | 2% | 5,679 |
|  | Other Backward Classes | 58% | 12% | 11% | 41% | 90% | 14 | 63.3 | 2% | 2,506 |
|  | Others | 67% | 13% | 13% | 44% | 90% | 12 | 66.1 | 3% | 5,252 |
|  |  |  |  |  |  |  |  |  |  |  |
| **Meghalaya** |  | 60% | 22% | 2% | 24% | 94% | 8 | 63.6 | 2% | 9,202 |
| Residence |  |  |  |  |  |  |  |  |  |  |
|  | Urban | 48% | 28% | 5% | 26% | 95% | 6 | 63.5 | 3% | 1,946 |
|  | Rural | 64% | 21% | 2% | 23% | 94% | 9 | 63.7 | 2% | 7,256 |
| Education |  |  |  |  |  |  |  |  |  |  |
|  | <5 years | 78% | 21% | 2% | 23% | 94% | 8 | 62.8 | 2% | 2,811 |
|  | 5-9 years | 57% | 23% | 2% | 23% | 95% | 8 | 63.4 | 2% | 3,330 |
|  | 10 years plus | 48% | 21% | 3% | 25% | 94% | 12 | 64.5 | 4% | 3,061 |
| Wealth status |  |  |  |  |  |  |  |  |  |  |
|  | Low | 70% | 18% | 2% | 25% | 94% | 8 | 72.6 | 1% | 3,068 |
|  | Middle | 57% | 24% | 2% | 23% | 95% | 8 | 64.3 | 3% | 4,783 |
|  | High | 48% | 27% | 5% | 22% | 95% | 12 | 53.9 | 5% | 1,351 |
| Caste |  |  |  |  |  |  |  |  |  |  |
|  | Scheduled caste/tribe | 60% | 20% | 2% | 24% | 95% | 8 | 71.5 | 3% | 8,639 |
|  | Other Backward Classes | 57% | 30% | 0% | 20% | 96% | 1 | 65.4 | 4% | 72 |
|  | Others | 70% | 38% | 6% | 22% | 93% | 9 | 53.9 | 0% | 461 |
|  |  |  |  |  |  |  |  |  |  |  |
|  |  | **Among all women, % currently married*** | **% using modern methods** | **% using tradition-**  **al methods** | **% with unmet need for modern methods** | **Average contra-**  **ceptive use effective-**  **ness** | **Average months of post-**  **partum infecund-**  **ability** | **Abortion**  **rate** | **% had non-contra-**  **ceptive**  **hyster-**  **ectomy** | **Number of Women: Unweighted Ns** |
| **Mizoram** |  | 53% | 35% | 0% | 20% | 97% | 4 | 63.6 | 5% | 12,279 |
| Residence |  |  |  |  |  |  |  |  |  |  |
|  | Urban | 47% | 38% | 0% | 20% | 97% | 4 | 61.2 | 6% | 6,198 |
|  | Rural | 62% | 32% | 0% | 21% | 97% | 5 | 66.0 | 3% | 6,081 |
| Education |  |  |  |  |  |  |  |  |  |  |
|  | <5 years | 78% | 27% | 0% | 18% | 98% | 5 | 56.7 | 4% | 2,016 |
|  | 5-9 years | 56% | 41% | 0% | 20% | 97% | 4 | 64.7 | 5% | 5,960 |
|  | 10 years plus | 40% | 33% | 0% | 22% | 97% | 5 | 69.4 | 4% | 4,303 |
| Wealth status |  |  |  |  |  |  |  |  |  |  |
|  | Low | 73% | 22% | 0% | 24% | 97% | 6 | 71.7 | 2% | 1,571 |
|  | Middle | 59% | 38% | 0% | 20% | 97% | 3 | 60.0 | 3% | 4,815 |
|  | High | 46% | 37% | 0% | 19% | 97% | 5 | 59.0 | 6% | 5,893 |
| Caste |  |  |  |  |  |  |  |  |  |  |
|  | Scheduled caste/tribe | 53% | 35% | 0% | 20% | 97% | 4 | 57.9 | 4% | 11,946 |
|  | Other Backward Classes | 57% | 45% | 0% | 21% | 97% | 7 | 61.0 | 7% | 259 |
|  | Others | 56% | 37% | 0% | 25% | 97% | 1 | 71.9 | 0% | 48 |
|  |  |  |  |  |  |  |  |  |  |  |
| **Nagaland** |  | 61% | 21% | 5% | 28% | 94% | 3 | 63.6 | 2% | 10,790 |
| Residence |  |  |  |  |  |  |  |  |  |  |
|  | Urban | 54% | 25% | 6% | 28% | 94% | 2 | 63.8 | 3% | 3,701 |
|  | Rural | 66% | 19% | 5% | 27% | 95% | 4 | 63.4 | 2% | 7,089 |
| Education |  |  |  |  |  |  |  |  |  |  |
|  | <5 years | 80% | 19% | 4% | 26% | 95% | 5 | 62.3 | 3% | 2,496 |
|  | 5-9 years | 64% | 23% | 5% | 26% | 95% | 3 | 59.5 | 2% | 4,927 |
|  | 10 years plus | 45% | 21% | 7% | 31% | 94% | 3 | 69.0 | 2% | 3,367 |
| Wealth status |  |  |  |  |  |  |  |  |  |  |
|  | Low | 73% | 17% | 4% | 29% | 95% | 4 | 72.1 | 1% | 3,688 |
|  | Middle | 60% | 24% | 5% | 26% | 95% | 3 | 57.5 | 3% | 4,762 |
|  | High | 50% | 23% | 8% | 29% | 93% | 2 | 61.2 | 4% | 2,340 |
| Caste |  |  |  |  |  |  |  |  |  |  |
|  | Scheduled caste/tribe | 60% | 21% | 5% | 27% | 95% | 3 | 66.2 | 2% | 10,365 |
|  |  | **Among all women, % currently married*** | **% using modern methods** | **% using tradition-**  **al methods** | **% with unmet need for modern methods** | **Average contra-**  **ceptive use effective-**  **ness** | **Average months of post-**  **partum infecund-**  **ability** | **Abortion**  **rate** | **% had non-contra-**  **ceptive**  **hyster-**  **ectomy** | **Number of Women: Unweighted Ns** |
|  | Other Backward Classes | 70% | 30% | 4% | 21% | 95% | 10 | 50.1 | 6% | 67 |
|  | Others | 74% | 32% | 6% | 31% | 93% | 8 | 74.5 | 6% | 319 |
|  |  |  |  |  |  |  |  |  |  |  |
| **Sikkim** |  | 63% | 46% | 1% | 23% | 97% | 11 | 63.6 | 3% | 5,293 |
| Residence |  |  |  |  |  |  |  |  |  |  |
|  | Urban | 63% | 36% | 1% | 26% | 96% | 13 | 69.4 | 3% | 1,369 |
|  | Rural | 63% | 51% | 1% | 21% | 97% | 9 | 57.8 | 3% | 3,924 |
| Education |  |  |  |  |  |  |  |  |  |  |
|  | <5 years | 86% | 54% | 1% | 17% | 98% | 12 | 47.3 | 4% | 1,168 |
|  | 5-9 years | 69% | 47% | 1% | 24% | 97% | 12 | 69.0 | 2% | 2,050 |
|  | 10 years plus | 46% | 36% | 1% | 26% | 96% | 8 | 74.5 | 2% | 2,075 |
| Wealth status |  |  |  |  |  |  |  |  |  |  |
|  | Low | 68% | 43% | 2% | 21% | 97% | 18 | 57.1 | 5% | 193 |
|  | Middle | 63% | 50% | 1% | 21% | 97% | 9 | 62.2 | 2% | 3,524 |
|  | High | 62% | 38% | 1% | 25% | 97% | 11 | 71.5 | 3% | 1,576 |
| Caste |  |  |  |  |  |  |  |  |  |  |
|  | Scheduled caste/tribe | 60% | 50% | 1% | 23% | 97% | 10 | 65.3 | 2% | 2,651 |
|  | Other Backward Classes | 65% | 48% | 0% | 21% | 97% | 9 | 59.8 | 2% | 1,427 |
|  | Others | 67% | 37% | 1% | 23% | 97% | 11 | 65.6 | 5% | 1,207 |
|  |  |  |  |  |  |  |  |  |  |  |
| **Tripura** |  | 76% | 43% | 21% | 32% | 91% | 14 | 63.6 | 2% | 4,804 |
| Residence |  |  |  |  |  |  |  |  |  |  |
|  | Urban | 74% | 43% | 24% | 35% | 91% | 18 | 65.2 | 1% | 1,375 |
|  | Rural | 77% | 43% | 20% | 31% | 91% | 13 | 62.0 | 2% | 3,429 |
| Education |  |  |  |  |  |  |  |  |  |  |
|  | <5 years | 86% | 43% | 20% | 29% | 92% | 14 | 56.6 | 2% | 1,228 |
|  | 5-9 years | 75% | 45% | 20% | 31% | 91% | 12 | 60.3 | 2% | 2,517 |
|  | 10 years plus | 66% | 38% | 26% | 39% | 90% | 19 | 73.9 | 1% | 1,059 |
| Wealth status |  |  |  |  |  |  |  |  |  |  |
|  | Low | 74% | 42% | 21% | 31% | 91% | 14 | 58.2 | 2% | 2,355 |
|  | Middle | 78% | 45% | 21% | 30% | 91% | 18 | 51.5 | 1% | 1,825 |
|  |  | **Among all women, % currently married*** | **% using modern methods** | **% using tradition-**  **al methods** | **% with unmet need for modern methods** | **Average contra-**  **ceptive use effective-**  **ness** | **Average months of post-**  **partum infecund-**  **ability** | **Abortion**  **rate** | **% had non-contra-**  **ceptive**  **hyster-**  **ectomy** | **Number of Women: Unweighted Ns** |
|  | High | 77% | 37% | 24% | 39% | 91% | 7 | 81.1 | 3% | 624 |
| Caste |  |  |  |  |  |  |  |  |  |  |
|  | Scheduled caste/tribe | 76% | 42% | 22% | 32% | 91% | 14 | 56.8 | 1% | 2,535 |
|  | Other Backward Classes | 75% | 44% | 24% | 34% | 91% | 18 | 59.6 | 2% | 880 |
|  | Others | 77% | 44% | 18% | 31% | 91% | 13 | 74.4 | 2% | 1,384 |
| **West** |  |  |  |  |  |  |  |  |  |  |
| **Goa** |  | 66% | 25% | 2% | 19% | 96% | 9 | 36.6 | 11% | 1,696 |
| Residence |  |  |  |  |  |  |  |  |  |  |
|  | Urban | 68% | 32% | 2% | 18% | 97% | 8 | 34.7 | 7% | 835 |
|  | Rural | 62% | 12% | 1% | 20% | 95% | 15 | 38.5 | 17% | 861 |
| Education |  |  |  |  |  |  |  |  |  |  |
|  | <5 years | 83% | 30% | 0% | 11% | 99% | 15 | 24.0 | 18% | 246 |
|  | 5-9 years | 69% | 25% | 1% | 21% | 97% | 8 | 43.8 | 11% | 478 |
|  | 10 years plus | 60% | 23% | 2% | 21% | 95% | 9 | 42.0 | 8% | 972 |
| Wealth status |  |  |  |  |  |  |  |  |  |  |
|  | Low | 64% | 12% | 1% | 22% | 98% | 8 | 43.0 | 15% | 67 |
|  | Middle | 65% | 29% | 0% | 15% | 98% | 19 | 29.3 | 14% | 390 |
|  | High | 66% | 24% | 2% | 20% | 96% | 8 | 37.6 | 9% | 1,239 |
| Caste |  |  |  |  |  |  |  |  |  |  |
|  | Scheduled caste/tribe | 63% | 28% | 0% | 16% | 98% | 5 | 32.8 | 11% | 299 |
|  | Other Backward Classes | 67% | 17% | 1% | 20% | 95% | 12 | 39.4 | 14% | 417 |
|  | Others | 66% | 27% | 2% | 20% | 96% | 9 | 37.6 | 9% | 980 |
|  |  |  |  |  |  |  |  |  |  |  |
| **Gujarat** |  | 74% | 43% | 4% | 21% | 97% | 4 | 47.6 | 4% | 22,932 |
| Residence |  |  |  |  |  |  |  |  |  |  |
|  | Urban | 74% | 41% | 6% | 25% | 96% | 2 | 51.6 | 3% | 8,202 |
|  | Rural | 74% | 45% | 2% | 18% | 98% | 5 | 43.6 | 5% | 14,730 |
| Education |  |  |  |  |  |  |  |  |  |  |
|  | <5 years | 88% | 51% | 2% | 13% | 99% | 7 | 31.6 | 6% | 7,530 |
|  | 5-9 years | 74% | 40% | 5% | 24% | 97% | 4 | 51.8 | 3% | 8,571 |
|  | 10 years plus | 62% | 37% | 5% | 27% | 95% | 1 | 59.4 | 3% | 6,831 |
|  |  | **Among all women, % currently married*** | **% using modern methods** | **% using tradition-**  **al methods** | **% with unmet need for modern methods** | **Average contra-**  **ceptive use effective-**  **ness** | **Average months of post-**  **partum infecund-**  **ability** | **Abortion**  **rate** | **% had non-contra-**  **ceptive**  **hyster-**  **ectomy** | **Number of Women: Unweighted Ns** |
| Wealth status |  |  |  |  |  |  |  |  |  |  |
|  | Low | 74% | 40% | 2% | 19% | 98% | 7 | 48.0 | 5% | 5,715 |
|  | Middle | 74% | 45% | 3% | 19% | 98% | 5 | 45.0 | 4% | 8,289 |
|  | High | 74% | 43% | 5% | 23% | 96% | 1 | 49.9 | 4% | 8,928 |
| Caste |  |  |  |  |  |  |  |  |  |  |
|  | Scheduled caste/tribe | 73% | 45% | 2% | 18% | 98% | 6 | 45.8 | 4% | 7,663 |
|  | Other Backward Classes | 75% | 43% | 4% | 21% | 97% | 4 | 49.1 | 4% | 9,536 |
|  | Others | 74% | 42% | 5% | 22% | 96% | 1 | 47.9 | 4% | 5,442 |
|  |  |  |  |  |  |  |  |  |  |  |
| **Maharashtra** |  | 73% | 63% | 2% | 12% | 98% | 6 | 36.6 | 2% | 29,460 |
| Residence |  |  |  |  |  |  |  |  |  |  |
|  | Urban | 69% | 61% | 3% | 14% | 97% | 5 | 41.1 | 1% | 10,983 |
|  | Rural | 77% | 64% | 1% | 10% | 98% | 6 | 32.2 | 3% | 18,477 |
| Education |  |  |  |  |  |  |  |  |  |  |
|  | <5 years | 85% | 74% | 1% | 6% | 99% | 9 | 19.0 | 3% | 7,293 |
|  | 5-9 years | 77% | 63% | 2% | 11% | 98% | 7 | 36.8 | 2% | 10,549 |
|  | 10 years plus | 63% | 53% | 3% | 17% | 96% | 4 | 54.1 | 1% | 11,618 |
| Wealth status |  |  |  |  |  |  |  |  |  |  |
|  | Low | 74% | 64% | 2% | 10% | 99% | 8 | 33.6 | 2% | 7,219 |
|  | Middle | 74% | 61% | 2% | 12% | 98% | 7 | 37.7 | 2% | 11,540 |
|  | High | 72% | 63% | 3% | 13% | 97% | 4 | 38.6 | 2% | 10,701 |
| Caste |  |  |  |  |  |  |  |  |  |  |
|  | Scheduled caste/tribe | 73% | 62% | 1% | 12% | 98% | 7 | 38.6 | 2% | 9,021 |
|  | Other Backward Classes | 74% | 65% | 2% | 11% | 98% | 5 | 33.9 | 2% | 8,835 |
|  | Others | 73% | 62% | 3% | 12% | 97% | 5 | 37.4 | 2% | 11,471 |
| **South** |  |  |  |  |  |  |  |  |  |  |
| **Andhra Pradesh** |  | 77% | 69% | 0% | 5% | 99% | 4 | 36.4 | 2% | 10,428 |
| Residence |  |  |  |  |  |  |  |  |  |  |
|  | Urban | 74% | 68% | 0% | 6% | 99% | 3 | 44.0 | 2% | 3,316 |
|  | Rural | 79% | 70% | 0% | 4% | 99% | 5 | 28.8 | 2% | 7,112 |
|  |  | **Among all women, % currently married*** | **% using modern methods** | **% using tradition-**  **al methods** | **% with unmet need for modern methods** | **Average contra-**  **ceptive use effective-**  **ness** | **Average months of post-**  **partum infecund-**  **ability** | **Abortion**  **rate** | **% had non-contra-**  **ceptive**  **hyster-**  **ectomy** | **Number of Women: Unweighted Ns** |
| Education |  |  |  |  |  |  |  |  |  |  |
|  | <5 years | 85% | 80% | 0% | 2% | 99% | 6 | 14.2 | 3% | 4,069 |
|  | 5-9 years | 84% | 70% | 0% | 4% | 99% | 4 | 29.2 | 2% | 2,754 |
|  | 10 years plus | 63% | 52% | 0% | 10% | 99% | 4 | 65.8 | 1% | 3,605 |
| Wealth status |  |  |  |  |  |  |  |  |  |  |
|  | Low | 76% | 65% | 0% | 4% | 99% | 6 | 31.8 | 2% | 1,441 |
|  | Middle | 78% | 71% | 0% | 4% | 99% | 5 | 29.2 | 2% | 5,329 |
|  | High | 77% | 68% | 0% | 6% | 99% | 3 | 48.2 | 2% | 3,658 |
| Caste |  |  |  |  |  |  |  |  |  |  |
|  | Scheduled caste/tribe | 76% | 65% | 0% | 5% | 99% | 7 | 35.7 | 3% | 2,587 |
|  | Other Backward Classes | 78% | 71% | 0% | 5% | 99% | 4 | 33.8 | 2% | 5,581 |
|  | Others | 77% | 71% | 0% | 5% | 99% | 1 | 39.7 | 3% | 2,221 |
|  |  |  |  |  |  |  |  |  |  |  |
| **Karnataka** |  | 73% | 51% | 0% | 11% | 99% | 6 | 36.4 | 4% | 26,291 |
| Residence |  |  |  |  |  |  |  |  |  |  |
|  | Urban | 71% | 47% | 1% | 13% | 99% | 7 | 42.8 | 4% | 8,998 |
|  | Rural | 74% | 54% | 0% | 9% | 99% | 6 | 30.0 | 4% | 17,293 |
| Education |  |  |  |  |  |  |  |  |  |  |
|  | <5 years | 83% | 64% | 0% | 4% | 99% | 9 | 15.2 | 5% | 8,648 |
|  | 5-9 years | 77% | 54% | 0% | 11% | 99% | 6 | 37.1 | 5% | 6,718 |
|  | 10 years plus | 64% | 39% | 1% | 17% | 98% | 5 | 56.9 | 3% | 10,925 |
| Wealth status |  |  |  |  |  |  |  |  |  |  |
|  | Low | 73% | 57% | 0% | 7% | 99% | 6 | 26.4 | 3% | 6,099 |
|  | Middle | 73% | 53% | 0% | 10% | 99% | 6 | 36.0 | 4% | 12,836 |
|  | High | 73% | 46% | 1% | 14% | 99% | 6 | 46.8 | 4% | 7,356 |
| Caste |  |  |  |  |  |  |  |  |  |  |
|  | Scheduled caste/tribe | 71% | 53% | 0% | 9% | 99% | 6 | 31.2 | 3% | 8,250 |
|  | Other Backward Classes | 73% | 52% | 1% | 11% | 99% | 5 | 38.0 | 4% | 12,906 |
|  | Others | 74% | 48% | 1% | 12% | 99% | 8 | 40.0 | 5% | 4,953 |
|  |  |  |  |  |  |  |  |  |  |  |
| **Kerala** |  | 74% | 50% | 3% | 17% | 98% | 5 | 36.4 | 2% | 11,033 |
|  |  | **Among all women, % currently married*** | **% using modern methods** | **% using tradition-**  **al methods** | **% with unmet need for modern methods** | **Average contra-**  **ceptive use effective-**  **ness** | **Average months of post-**  **partum infecund-**  **ability** | **Abortion**  **rate** | **% had non-contra-**  **ceptive**  **hyster-**  **ectomy** | **Number of Women: Unweighted Ns** |
| Residence |  |  |  |  |  |  |  |  |  |  |
|  | Urban | 75% | 51% | 3% | 17% | 98% | 4 | 37.9 | 2% | 4,187 |
|  | Rural | 73% | 50% | 3% | 16% | 98% | 8 | 34.9 | 2% | 6,846 |
| Education |  |  |  |  |  |  |  |  |  |  |
|  | <5 years | 79% | 63% | 2% | 6% | 99% | 6 | 15.0 | 4% | 437 |
|  | 5-9 years | 84% | 59% | 2% | 11% | 99% | 8 | 32.0 | 3% | 2,743 |
|  | 10 years plus | 70% | 46% | 3% | 19% | 98% | 5 | 62.3 | 2% | 7,853 |
| Wealth status |  |  |  |  |  |  |  |  |  |  |
|  | Low | 65% | 57% | 2% | 11% | 99% | 24 | 27.4 | 3% | 247 |
|  | Middle | 73% | 55% | 3% | 15% | 98% | 8 | 37.6 | 2% | 3,066 |
|  | High | 74% | 48% | 3% | 17% | 98% | 5 | 44.2 | 2% | 7,720 |
| Caste |  |  |  |  |  |  |  |  |  |  |
|  | Scheduled caste/tribe | 72% | 55% | 2% | 14% | 98% | 18 | 32.1 | 2% | 1,372 |
|  | Other Backward Classes | 74% | 50% | 3% | 17% | 98% | 6 | 38.4 | 2% | 5,937 |
|  | Others | 74% | 49% | 3% | 17% | 98% | 4 | 38.7 | 3% | 3,683 |
|  |  |  |  |  |  |  |  |  |  |  |
| **Tamil Nadu** |  | 73% | 53% | 1% | 11% | 99% | 3 | 32.8 | 4% | 28,820 |
| Residence |  |  |  |  |  |  |  |  |  |  |
|  | Urban | 73% | 53% | 1% | 11% | 99% | 2 | 34.4 | 4% | 12,806 |
|  | Rural | 73% | 52% | 1% | 10% | 99% | 4 | 31.1 | 5% | 16,014 |
| Education |  |  |  |  |  |  |  |  |  |  |
|  | <5 years | 85% | 61% | 0% | 4% | 99% | 7 | 12.4 | 7% | 5,929 |
|  | 5-9 years | 83% | 57% | 1% | 9% | 99% | 4 | 32.3 | 5% | 8,622 |
|  | 10 years plus | 63% | 45% | 1% | 16% | 99% | 3 | 53.7 | 3% | 14,269 |
| Wealth status |  |  |  |  |  |  |  |  |  |  |
|  | Low | 69% | 51% | 1% | 9% | 99% | 5 | 28.7 | 6% | 4,189 |
|  | Middle | 73% | 53% | 1% | 11% | 99% | 4 | 33.3 | 5% | 13,990 |
|  | High | 74% | 53% | 1% | 11% | 99% | 2 | 36.3 | 4% | 10,641 |
| Caste |  |  |  |  |  |  |  |  |  |  |
|  | Scheduled caste/tribe | 71% | 53% | 1% | 10% | 99% | 5 | 31.2 | 5% | 9,034 |
|  | Other Backward Classes | 74% | 52% | 1% | 11% | 99% | 3 | 36.0 | 4% | 19,266 |
|  |  | **Among all women, % currently married*** | **% using modern methods** | **% using tradition-**  **al methods** | **% with unmet need for modern methods** | **Average contra-**  **ceptive use effective-**  **ness** | **Average months of post-**  **partum infecund-**  **ability** | **Abortion**  **rate** | **% had non-contra-**  **ceptive**  **hyster-**  **ectomy** | **Number of Women: Unweighted Ns** |
|  | Others | 76% | 53% | 1% | 10% | 99% | 5 | 31.2 | 5% | 509 |
|  |  |  |  |  |  |  |  |  |  |  |
| **Telangana** |  | 74% | 57% | 0% | 8% | 99% | 6 | 36.4 | 5% | 7,567 |
| Residence |  |  |  |  |  |  |  |  |  |  |
|  | Urban | 73% | 58% | 1% | 9% | 99% | 4 | 43.5 | 3% | 2,775 |
|  | Rural | 76% | 56% | 0% | 6% | 99% | 7 | 29.3 | 7% | 4,792 |
| Education |  |  |  |  |  |  |  |  |  |  |
|  | <5 years | 84% | 66% | 0% | 4% | 99% | 8 | 17.0 | 9% | 3,128 |
|  | 5-9 years | 77% | 58% | 0% | 7% | 99% | 6 | 35.1 | 5% | 1,512 |
|  | 10 years plus | 65% | 47% | 1% | 12% | 99% | 4 | 57.1 | 1% | 2,927 |
| Wealth status |  |  |  |  |  |  |  |  |  |  |
|  | Low | 74% | 51% | 0% | 6% | 100% | 7 | 32.2 | 5% | 1,550 |
|  | Middle | 74% | 57% | 0% | 6% | 99% | 7 | 31.8 | 6% | 3,501 |
|  | High | 75% | 59% | 1% | 10% | 99% | 3 | 45.2 | 4% | 2,516 |
| Caste |  |  |  |  |  |  |  |  |  |  |
|  | Scheduled caste/tribe | 72% | 54% | 0% | 6% | 99% | 6 | 31.3 | 6% | 2,200 |
|  | Other Backward Classes | 75% | 57% | 0% | 8% | 99% | 6 | 35.5 | 5% | 4,324 |
|  | Others | 75% | 60% | 1% | 9% | 99% | 3 | 42.4 | 4% | 988 |
