# Supplementary material for: Key drivers of fertility levels and differentials in India, at the national, state and population subgroup levels, 2015–2016: An application of Bongaarts’ proximate determinants model
Source: PLoS One. 2022 Feb 7;17(2):e0263532. doi: 10.1371/journal.pone.0263532 (PMC8820640; doi:10.1371/journal.pone.0263532)
Supplement: S2 Table — (DOCX) [file pone.0263532.s002.docx]

**S2 Table: Estimates of the percent contribution of four key proximate determinants to fertility reduction by age-group, actual ASFR and Number of Women: By state, India 2015-16**

|  |  |  | **% contribution of determinant to  fertility reduction from total fecundity§** | | | |  |  |  |  |  |  |  |  |
| --- | --- | --- | --- | --- | --- | --- | --- | --- | --- | --- | --- | --- | --- | --- |
| **Region** | **State** | **Age group** | **Marriage** | **Contra- ception** | **Abortion** | **Postpartum Infecund- ability** | **Age-specific fertility rate** | **Number of Women: Unweighted Ns** |  |  |  |  |  |  |
| North | Haryana | 15-19 | 83.2 | 3.1 | 9.3 | 4.4 | 41 | 3,358 |  |  |  |  |  |  |
|  |  | 20-24 | 40.2 | 21.5 | 22.4 | 16.0 | 189 | 4,327 |  |  |  |  |  |  |
|  |  | 25-29 | 5.2 | 50.0 | 26.3 | 18.5 | 123 | 3,872 |  |  |  |  |  |  |
|  |  | 30-34 | 1.6 | 58.2 | 27.7 | 12.5 | 40 | 3,024 |  |  |  |  |  |  |
|  |  | 35-39 | 1.4 | 57.7 | 26.2 | 14.8 | 12 | 2,587 |  |  |  |  |  |  |
|  |  | 40-44 | 1.8 | 55.2 | 32.9 | {10.1} | 5 | 2,331 |  |  |  |  |  |  |
|  |  | 45-49 | 2.8 | 53.1 | 44.1 | b | 2 | 2,155 |  |  |  |  |  |  |
|  |  |  |  |  |  |  |  |  |  |  |  |  |  |  |
| North | Himachal Pradesh | 15-19 | 84.0 | 1.7 | 12.0 | {2.2} | 25 | 1,358 |  |  |  |  |  |  |
|  |  | 20-24 | 53.4 | 16.7 | 24.0 | 5.9 | 154 | 1,393 |  |  |  |  |  |  |
|  |  | 25-29 | 11.7 | 44.6 | 36.7 | 7.0 | 130 | 1,574 |  |  |  |  |  |  |
|  |  | 30-34 | 3.1 | 55.8 | 36.8 | 4.4 | 50 | 1,587 |  |  |  |  |  |  |
|  |  | 35-39 | 2.0 | 51.5 | 45.6 | 0.9 | 11 | 1,470 |  |  |  |  |  |  |
|  |  | 40-44 | 1.7 | 46.8 | 51.5 | b | 4 | 1,373 |  |  |  |  |  |  |
|  |  | 45-49 | 2.5 | 46.6 | 34.2 | b | 1 | 1,174 |  |  |  |  |  |  |
|  |  |  |  |  |  |  |  |  |  |  |  |  |  |  |
| North | Jammu & Kashmir | 15-19 | 82.6 | 1.4 | 12.9 | 3.1 | 19 | 4,156 |  |  |  |  |  |  |
|  |  | 20-24 | 61.9 | 8.5 | 18.6 | 11.1 | 113 | 4,283 |  |  |  |  |  |  |
|  |  | 25-29 | 28.3 | 28.2 | 22.2 | 21.3 | 142 | 4,254 |  |  |  |  |  |  |
|  |  | 30-34 | 7.4 | 53.0 | 21.1 | 18.5 | 90 | 3,347 |  |  |  |  |  |  |
|  |  | 35-39 | 2.3 | 57.2 | 24.8 | 15.7 | 28 | 3,185 |  |  |  |  |  |  |
|  |  | 40-44 | 1.6 | 57.0 | 34.4 | 7.0 | 7 | 2,425 |  |  |  |  |  |  |
|  |  | 45-49 | 2.2 | 56.9 | 34.9 | {6.0} | 3 | 2,150 |  |  |  |  |  |  |
|  |  |  |  |  |  |  |  |  |  |  |  |  |  |  |
| North | Punjab | 15-19 | 80.9 | 3.4 | 12.5 | 3.2 | 20 | 2,670 |  |  |  |  |  |  |
|  |  | 20-24 | 52.3 | 19.4 | 21.0 | 7.3 | 123 | 3,250 |  |  |  |  |  |  |
|  |  | 25-29 | 14.4 | 53.3 | 27.5 | 4.9 | 120 | 3,354 |  |  |  |  |  |  |
|  |  | 30-34 | 2.3 | 64.8 | 24.6 | 8.3 | 45 | 2,944 |  |  |  |  |  |  |
|  |  | 35-39 | 1.5 | 65.1 | 30.1 | 3.3 | 14 | 2,716 |  |  |  |  |  |  |
|  |  | 40-44 | 1.7 | 48.0 | 49.8 | {.5} | 2 | 2,366 |  |  |  |  |  |  |
|  |  | 45-49 | c | c | c | c | c | 2,184 |  |  |  |  |  |  |
|  |  |  |  |  |  |  |  |  |  |  |  |  |  |  |
| North | Rajasthan | 15-19 | 83.1 | 3.3 | 10.3 | 3.2 | 46 | 8,132 |  |  |  |  |  |  |
|  |  | 20-24 | 38.8 | 22.2 | 25.1 | 13.9 | 211 | 8,201 |  |  |  |  |  |  |
|  |  | 25-29 | 5.8 | 52.7 | 31.0 | 10.6 | 140 | 6,632 |  |  |  |  |  |  |
|  |  | 30-34 | 1.7 | 64.1 | 26.2 | 8.0 | 55 | 5,796 |  |  |  |  |  |  |
|  |  | 35-39 | 1.5 | 62.6 | 27.8 | 8.1 | 18 | 5,070 |  |  |  |  |  |  |
|  |  | 40-44 | 1.7 | 60.2 | 32.8 | 5.3 | 7 | 4,207 |  |  |  |  |  |  |
|  |  | 45-49 | 2.5 | 56.8 | 32.8 | {7.9} | 3 | 3,927 |  |  |  |  |  |  |
|  |  |  |  |  |  |  |  |  |  |  |  |  |  |  |
| North | Uttarakhand | 15-19 | 84.2 | 1.2 | 12.4 | 2.2 | 23 | 3,285 |  |  |  |  |  |  |
|  |  | 20-24 | 51.5 | 13.4 | 21.0 | 14.0 | 175 | 3,108 |  |  |  |  |  |  |
|  |  | 25-29 | 13.2 | 39.3 | 26.8 | 20.7 | 137 | 2,804 |  |  |  |  |  |  |
|  |  | 30-34 | 2.9 | 53.1 | 27.5 | 16.6 | 58 | 2,337 |  |  |  |  |  |  |
|  |  | 35-39 | 2.3 | 54.3 | 37.2 | 6.2 | 16 | 2,200 |  |  |  |  |  |  |
|  |  | 40-44 | 2.4 | 44.8 | 43.7 | {9.1} | 3 | 1,786 |  |  |  |  |  |  |
|  |  | 45-49 | 3.6 | 56.0 | 37.5 | b | 3 | 1,780 |  |  |  |  |  |  |
|  |  |  |  |  |  |  |  |  |  |  |  |  |  |  |
| Central | Chhattisgarh | 15-19 | 84.6 | 1.3 | 10.1 | 3.9 | 36 | 4,651 |  |  |  |  |  |  |
|  |  | 20-24 | 47.1 | 11.7 | 24.6 | 16.6 | 196 | 4,604 |  |  |  |  |  |  |
|  |  | 25-29 | 11.2 | 39.1 | 29.3 | 20.3 | 133 | 4,366 |  |  |  |  |  |  |
|  |  | 30-34 | 4.1 | 55.0 | 23.4 | 17.5 | 58 | 3,273 |  |  |  |  |  |  |
|  |  | 35-39 | 3.2 | 56.6 | 26.3 | 13.9 | 18 | 2,883 |  |  |  |  |  |  |
|  |  | 40-44 | 2.7 | 52.8 | 32.0 | 12.5 | 4 | 2,629 |  |  |  |  |  |  |
|  |  | 45-49 | 2.7 | 40.3 | 43.4 | b | 1 | 2,766 |  |  |  |  |  |  |
|  |  |  |  |  |  |  |  |  |  |  |  |  |  |  |
| Central | Madhya Pradesh | 15-19 | 82.5 | 1.7 | 8.9 | 6.9 | 53 | 11,735 |  |  |  |  |  |  |
|  |  | 20-24 | 36.2 | 14.1 | 22.5 | 27.1 | 220 | 11,618 |  |  |  |  |  |  |
|  |  | 25-29 | 6.4 | 40.1 | 30.0 | 23.5 | 126 | 9,975 |  |  |  |  |  |  |
|  |  | 30-34 | 2.4 | 50.9 | 31.9 | 14.8 | 46 | 8,428 |  |  |  |  |  |  |
|  |  | 35-39 | 2.1 | 48.8 | 37.4 | 11.7 | 14 | 7,614 |  |  |  |  |  |  |
|  |  | 40-44 | 2.2 | 43.6 | 46.7 | 7.5 | 4 | 6,772 |  |  |  |  |  |  |
|  |  | 45-49 | 2.5 | 49.0 | 43.3 | {5.2} | 1 | 6,661 |  |  |  |  |  |  |
|  |  |  |  |  |  |  |  |  |  |  |  |  |  |  |
| Central | Uttar Pradesh | 15-19 | 82.0 | 1.9 | 13.7 | 2.4 | 28 | 22,032 |  |  |  |  |  |  |
|  |  | 20-24 | 48.0 | 13.3 | 23.6 | 15.1 | 198 | 18,719 |  |  |  |  |  |  |
|  |  | 25-29 | 10.3 | 37.1 | 30.9 | 21.6 | 186 | 14,952 |  |  |  |  |  |  |
|  |  | 30-34 | 2.6 | 51.1 | 28.6 | 17.6 | 87 | 12,204 |  |  |  |  |  |  |
|  |  | 35-39 | 2.4 | 53.5 | 29.3 | 14.8 | 36 | 11,227 |  |  |  |  |  |  |
|  |  | 40-44 | 2.5 | 44.6 | 37.2 | 15.8 | 9 | 9,714 |  |  |  |  |  |  |
|  |  | 45-49 | 3.1 | 39.7 | 36.6 | 20.7 | 3 | 8,813 |  |  |  |  |  |  |
|  |  |  |  |  |  |  |  |  |  |  |  |  |  |  |
| East | Bihar | 15-19 | 82.6 | 0.5 | 7.5 | 9.4 | 77 | 10,088 |  |  |  |  |  |  |
|  |  | 20-24 | 29.4 | 5.6 | 21.5 | 43.5 | 273 | 7,897 |  |  |  |  |  |  |
|  |  | 25-29 | 5.3 | 22.9 | 29.2 | 42.6 | 185 | 7,612 |  |  |  |  |  |  |
|  |  | 30-34 | 2.4 | 35.1 | 24.5 | 37.9 | 91 | 6,170 |  |  |  |  |  |  |
|  |  | 35-39 | 3.0 | 35.2 | 27.1 | 34.7 | 38 | 5,463 |  |  |  |  |  |  |
|  |  | 40-44 | 4.0 | 31.2 | 36.9 | 28.0 | 12 | 4,346 |  |  |  |  |  |  |
|  |  | 45-49 | 4.5 | 31.2 | 40.3 | 24.0 | 5 | 4,236 |  |  |  |  |  |  |
|  |  |  |  |  |  |  |  |  |  |  |  |  |  |  |
| East | Jharkhand | 15-19 | 81.5 | 1.7 | 7.7 | 9.0 | 77 | 5,669 |  |  |  |  |  |  |
|  |  | 20-24 | 36.3 | 10.1 | 21.3 | 32.3 | 214 | 5,310 |  |  |  |  |  |  |
|  |  | 25-29 | 9.2 | 29.5 | 24.8 | 36.6 | 131 | 4,785 |  |  |  |  |  |  |
|  |  | 30-34 | 3.7 | 42.5 | 25.5 | 28.3 | 58 | 4,057 |  |  |  |  |  |  |
|  |  | 35-39 | 3.3 | 44.8 | 33.8 | 18.1 | 19 | 3,587 |  |  |  |  |  |  |
|  |  | 40-44 | 3.9 | 41.8 | 42.1 | 12.1 | 7 | 2,917 |  |  |  |  |  |  |
|  |  | 45-49 | 4.1 | 41.5 | 33.6 | {20.8} | 2 | 2,721 |  |  |  |  |  |  |
|  |  |  |  |  |  |  |  |  |  |  |  |  |  |  |
| East | Odisha | 15-19 | 80.3 | 4.2 | 9.0 | 6.5 | 46 | 5,580 |  |  |  |  |  |  |
|  |  | 20-24 | 38.8 | 15.9 | 17.9 | 27.4 | 156 | 5,858 |  |  |  |  |  |  |
|  |  | 25-29 | 10.5 | 33.4 | 19.2 | 36.9 | 128 | 5,446 |  |  |  |  |  |  |
|  |  | 30-34 | 4.6 | 44.7 | 18.2 | 32.6 | 55 | 4,725 |  |  |  |  |  |  |
|  |  | 35-39 | 3.6 | 45.2 | 22.1 | 29.2 | 18 | 4,361 |  |  |  |  |  |  |
|  |  | 40-44 | 3.7 | 44.1 | 34.4 | 17.8 | 5 | 3,917 |  |  |  |  |  |  |
|  |  | 45-49 | 3.1 | 50.6 | 34.9 | {11.3} | 2 | 3,834 |  |  |  |  |  |  |
|  |  |  |  |  |  |  |  |  |  |  |  |  |  |  |
| East | West Bengal | 15-19 | 71.3 | 12.0 | 7.1 | 9.6 | 90 | 3,016 |  |  |  |  |  |  |
|  |  | 20-24 | 18.5 | 30.2 | 14.9 | 36.4 | 147 | 3,157 |  |  |  |  |  |  |
|  |  | 25-29 | 5.2 | 45.7 | 20.0 | 29.1 | 77 | 2,781 |  |  |  |  |  |  |
|  |  | 30-34 | 2.1 | 55.1 | 18.3 | 24.5 | 31 | 2,436 |  |  |  |  |  |  |
|  |  | 35-39 | 2.1 | 50.7 | 29.2 | 18.1 | 7 | 2,257 |  |  |  |  |  |  |
|  |  | 40-44 | 2.0 | 43.5 | 37.7 | b | 1 | 2,018 |  |  |  |  |  |  |
|  |  | 45-49 | 2.0 | 35.1 | 50.0 | b | 0* | 2,003 |  |  |  |  |  |  |
|  |  |  |  |  |  |  |  |  |  |  |  |  |  |  |
| Northeast | Arunachal Pradesh | 15-19 | 80.1 | 3.5 | 9.4 | 6.9 | 56 | 2,233 |  |  |  |  |  |  |
|  |  | 20-24 | 41.6 | 9.5 | 24.8 | 24.1 | 130 | 2,212 |  |  |  |  |  |  |
|  |  | 25-29 | 15.5 | 20.8 | 31.6 | 32.0 | 116 | 2,558 |  |  |  |  |  |  |
|  |  | 30-34 | 7.3 | 31.1 | 31.4 | 30.2 | 67 | 2,082 |  |  |  |  |  |  |
|  |  | 35-39 | 4.1 | 33.9 | 31.4 | 30.7 | 32 | 2,079 |  |  |  |  |  |  |
|  |  | 40-44 | 6.0 | 30.3 | 35.9 | 27.7 | 16 | 1,523 |  |  |  |  |  |  |
|  |  | 45-49 | 7.8 | 20.0 | 54.9 | {17.2} | 4 | 1,607 |  |  |  |  |  |  |
|  |  |  |  |  |  |  |  |  |  |  |  |  |  |  |
| Northeast | Assam | 15-19 | 74.8 | 6.6 | 8.6 | 10.0 | 72 | 4,671 |  |  |  |  |  |  |
|  |  | 20-24 | 29.1 | 21.7 | 20.7 | 28.5 | 151 | 4,963 |  |  |  |  |  |  |
|  |  | 25-29 | 11.0 | 37.2 | 24.4 | 27.4 | 120 | 4,871 |  |  |  |  |  |  |
|  |  | 30-34 | 5.6 | 44.6 | 21.6 | 28.2 | 65 | 3,964 |  |  |  |  |  |  |
|  |  | 35-39 | 4.8 | 43.1 | 27.2 | 24.9 | 25 | 3,875 |  |  |  |  |  |  |
|  |  | 40-44 | 5.5 | 33.7 | 50.3 | 10.5 | 7 | 3,011 |  |  |  |  |  |  |
|  |  | 45-49 | 7.5 | 18.8 | 56.4 | {17.4} | 2 | 3,092 |  |  |  |  |  |  |
|  |  |  |  |  |  |  |  |  |  |  |  |  |  |  |
| Northeast | Manipur | 15-19 | 83.6 | 1.5 | 10.6 | 4.3 | 43 | 2,071 |  |  |  |  |  |  |
|  |  | 20-24 | 53.5 | 8.0 | 23.9 | 14.6 | 144 | 2,103 |  |  |  |  |  |  |
|  |  | 25-29 | 29.0 | 14.9 | 26.9 | 29.3 | 146 | 2,263 |  |  |  |  |  |  |
|  |  | 30-34 | 18.7 | 25.3 | 27.1 | 28.8 | 117 | 2,106 |  |  |  |  |  |  |
|  |  | 35-39 | 14.0 | 27.9 | 31.9 | 26.2 | 56 | 2,016 |  |  |  |  |  |  |
|  |  | 40-44 | 9.8 | 16.7 | 31.7 | 41.8 | 15 | 1,588 |  |  |  |  |  |  |
|  |  | 45-49 | 5.9 | 7.5 | 86.6 | b | 1 | 1,446 |  |  |  |  |  |  |
|  |  |  |  |  |  |  |  |  |  |  |  |  |  |  |
| Northeast | Meghalaya | 15-19 | 83.9 | 1.4 | 9.0 | 5.7 | 48 | 1,829 |  |  |  |  |  |  |
|  |  | 20-24 | 54.5 | 5.5 | 20.6 | 19.4 | 151 | 1,626 |  |  |  |  |  |  |
|  |  | 25-29 | 27.0 | 17.7 | 26.9 | 28.4 | 165 | 1,660 |  |  |  |  |  |  |
|  |  | 30-34 | 15.7 | 29.4 | 28.1 | 26.8 | 123 | 1,208 |  |  |  |  |  |  |
|  |  | 35-39 | 12.9 | 36.8 | 23.5 | 26.8 | 83 | 1,118 |  |  |  |  |  |  |
|  |  | 40-44 | 15.3 | 33.0 | 35.0 | 16.7 | 30 | 857 |  |  |  |  |  |  |
|  |  | 45-49 | 13.6 | 14.9 | 30.2 | {41.3} | 9 | 904 |  |  |  |  |  |  |
|  |  |  |  |  |  |  |  |  |  |  |  |  |  |  |
| Northeast | Mizoram | 15-19 | 85.7 | 1.4 | 10.6 | 2.3 | 38 | 1,952 |  |  |  |  |  |  |
|  |  | 20-24 | 62.8 | 9.0 | 21.8 | 6.4 | 124 | 1,850 |  |  |  |  |  |  |
|  |  | 25-29 | 41.3 | 18.7 | 26.7 | 13.3 | 129 | 2,163 |  |  |  |  |  |  |
|  |  | 30-34 | 22.6 | 33.7 | 31.3 | 12.4 | 95 | 1,965 |  |  |  |  |  |  |
|  |  | 35-39 | 20.3 | 36.5 | 32.6 | 10.6 | 49 | 1,806 |  |  |  |  |  |  |
|  |  | 40-44 | 13.5 | 32.3 | 32.9 | 21.3 | 16 | 1,426 |  |  |  |  |  |  |
|  |  | 45-49 | 8.4 | 27.0 | 62.1 | b | 2 | 1,117 |  |  |  |  |  |  |
|  |  |  |  |  |  |  |  |  |  |  |  |  |  |  |
| Northeast | Nagaland | 15-19 | 85.8 | 0.4 | 9.8 | 3.9 | 42 | 1,715 |  |  |  |  |  |  |
|  |  | 20-24 | 58.9 | 5.7 | 21.7 | 13.7 | 150 | 1,691 |  |  |  |  |  |  |
|  |  | 25-29 | 40.8 | 16.2 | 36.4 | 6.7 | 154 | 1,866 |  |  |  |  |  |  |
|  |  | 30-34 | 23.1 | 34.9 | 39.5 | 2.4 | 110 | 1,655 |  |  |  |  |  |  |
|  |  | 35-39 | 14.8 | 40.6 | 38.3 | 6.4 | 63 | 1,492 |  |  |  |  |  |  |
|  |  | 40-44 | 10.0 | 36.1 | 53.9 | 0.0 | 21 | 1,168 |  |  |  |  |  |  |
|  |  | 45-49 | 9.5 | 28.3 | 62.1 | {0} | 8 | 1,203 |  |  |  |  |  |  |
|  |  |  |  |  |  |  |  |  |  |  |  |  |  |  |
| Northeast | Sikkim | 15-19 | a | a | a | b | 22 | 853 |  |  |  |  |  |  |
|  |  | 20-24 | 48.5 | 10.3 | 26.5 | 14.7 | 79 | 842 |  |  |  |  |  |  |
|  |  | 25-29 | 19.6 | 23.2 | 31.1 | 26.1 | 70 | 1,039 |  |  |  |  |  |  |
|  |  | 30-34 | 9.4 | 34.5 | 31.3 | 24.8 | 46 | 818 |  |  |  |  |  |  |
|  |  | 35-39 | 6.4 | 37.9 | 40.4 | 15.3 | 17 | 752 |  |  |  |  |  |  |
|  |  | 40-44 | 2.4 | 24.2 | 56.8 | b | 1 | 515 |  |  |  |  |  |  |
|  |  | 45-49 | c | c | c | c | c | 474 |  |  |  |  |  |  |
|  |  |  |  |  |  |  |  |  |  |  |  |  |  |  |
| Northeast | Tripura | 15-19 | 75.3 | 7.6 | 8.4 | 8.7 | 82 | 746 |  |  |  |  |  |  |
|  |  | 20-24 | 22.9 | 21.4 | 16.9 | 38.9 | 118 | 747 |  |  |  |  |  |  |
|  |  | 25-29 | 7.5 | 39.3 | 27.3 | 25.9 | 83 | 886 |  |  |  |  |  |  |
|  |  | 30-34 | 3.9 | 50.2 | 21.1 | 24.8 | 36 | 673 |  |  |  |  |  |  |
|  |  | 35-39 | 2.1 | 49.2 | 28.6 | {20.1} | 15 | 654 |  |  |  |  |  |  |
|  |  | 40-44 | 3.2 | 28.6 | 49.8 | b | 1 | 556 |  |  |  |  |  |  |
|  |  | 45-49 | 2.9 | 25.7 | 68.4 | b | 0* | 542 |  |  |  |  |  |  |
|  |  |  |  |  |  |  |  |  |  |  |  |  |  |  |
| West | Goa | 15-19 | a | a | a | b | 16 | 235 |  |  |  |  |  |  |
|  |  | 20-24 | [60.7] | [5.9] | [14.9] | {18.5} | 74 | 238 |  |  |  |  |  |  |
|  |  | 25-29 | 25.8 | 21.8 | 19.3 | 33.1 | 122 | 234 |  |  |  |  |  |  |
|  |  | 30-34 | 11.5 | 34.9 | 29.4 | 24.3 | 74 | 244 |  |  |  |  |  |  |
|  |  | 35-39 | 9.4 | 30.5 | 29.6 | {30.5} | 36 | 274 |  |  |  |  |  |  |
|  |  | 40-44 | 7.1 | 18.8 | 37.1 | b | 6 | 266 |  |  |  |  |  |  |
|  |  | 45-49 | 9.3 | 23.5 | 67.1 | b | 4 | 205 |  |  |  |  |  |  |
|  |  |  |  |  |  |  |  |  |  |  |  |  |  |  |
| West | Gujarat | 15-19 | 81.3 | 2.8 | 9.2 | 6.7 | 41 | 3,782 |  |  |  |  |  |  |
|  |  | 20-24 | 49.0 | 13.9 | 25.0 | 12.2 | 170 | 3,865 |  |  |  |  |  |  |
|  |  | 25-29 | 10.9 | 41.3 | 35.5 | 12.3 | 131 | 3,497 |  |  |  |  |  |  |
|  |  | 30-34 | 3.9 | 49.9 | 44.5 | 1.7 | 45 | 3,421 |  |  |  |  |  |  |
|  |  | 35-39 | 2.9 | 46.6 | 43.3 | 7.2 | 14 | 3,092 |  |  |  |  |  |  |
|  |  | 40-44 | 2.5 | 38.1 | 54.9 | {4.4} | 2 | 2,900 |  |  |  |  |  |  |
|  |  | 45-49 | 2.4 | 44.9 | 45.1 | b | 1 | 2,375 |  |  |  |  |  |  |
|  |  |  |  |  |  |  |  |  |  |  |  |  |  |  |
| West | Maharashtra | 15-19 | 85.5 | 2.3 | 6.4 | 5.7 | 59 | 4,753 |  |  |  |  |  |  |
|  |  | 20-24 | 44.9 | 21.1 | 17.6 | 16.3 | 179 | 5,036 |  |  |  |  |  |  |
|  |  | 25-29 | 10.0 | 49.4 | 25.1 | 15.5 | 96 | 4,911 |  |  |  |  |  |  |
|  |  | 30-34 | 3.6 | 59.8 | 26.2 | 10.4 | 30 | 4,097 |  |  |  |  |  |  |
|  |  | 35-39 | 2.9 | 59.1 | 23.5 | 14.5 | 9 | 4,028 |  |  |  |  |  |  |
|  |  | 40-44 | 2.6 | 46.2 | 42.0 | {9.1} | 1 | 3,645 |  |  |  |  |  |  |
|  |  | 45-49 | c | c | c | c | c | 2,990 |  |  |  |  |  |  |
|  |  |  |  |  |  |  |  |  |  |  |  |  |  |  |
| South | Andhra Pradesh | 15-19 | 86.5 | 0.9 | 5.6 | 7.0 | 83 | 1,348 |  |  |  |  |  |  |
|  |  | 20-24 | 37.9 | 26.6 | 21.3 | 14.2 | 184 | 1,741 |  |  |  |  |  |  |
|  |  | 25-29 | 6.6 | 57.8 | 28.0 | 7.6 | 75 | 1,721 |  |  |  |  |  |  |
|  |  | 30-34 | 3.0 | 60.6 | 28.3 | 8.1 | 19 | 1,441 |  |  |  |  |  |  |
|  |  | 35-39 | 3.0 | 53.9 | 40.3 | {2.8} | 4 | 1,550 |  |  |  |  |  |  |
|  |  | 40-44 | 3.1 | 46.6 | 46.6 | b | 0* | 1,211 |  |  |  |  |  |  |
|  |  | 45-49 | c | c | c | c | c | 1,416 |  |  |  |  |  |  |
|  |  |  |  |  |  |  |  |  |  |  |  |  |  |  |
| South | Karnataka | 15-19 | 88.2 | 0.9 | 7.5 | 3.4 | 51 | 3,903 |  |  |  |  |  |  |
|  |  | 20-24 | 47.1 | 13.1 | 18.6 | 21.2 | 167 | 4,331 |  |  |  |  |  |  |
|  |  | 25-29 | 13.3 | 38.3 | 26.8 | 21.6 | 102 | 4,490 |  |  |  |  |  |  |
|  |  | 30-34 | 5.3 | 50.1 | 33.5 | 11.1 | 31 | 3,837 |  |  |  |  |  |  |
|  |  | 35-39 | 4.1 | 50.2 | 44.8 | 0.9 | 8 | 3,705 |  |  |  |  |  |  |
|  |  | 40-44 | 3.4 | 37.8 | 52.8 | b | 1 | 3,203 |  |  |  |  |  |  |
|  |  | 45-49 | 3.7 | 37.9 | 58.5 | b | 0* | 2,822 |  |  |  |  |  |  |
|  |  |  |  |  |  |  |  |  |  |  |  |  |  |  |
| South | Kerala | 15-19 | [81.6] | [2.8] | [8.5] | b | 21 | 1,484 |  |  |  |  |  |  |
|  |  | 20-24 | 57.2 | 5.7 | 18.9 | 18.3 | 110 | 1,533 |  |  |  |  |  |  |
|  |  | 25-29 | 16.6 | 34.4 | 35.1 | 13.9 | 117 | 1,617 |  |  |  |  |  |  |
|  |  | 30-34 | 3.0 | 49.7 | 28.0 | 19.4 | 48 | 1,521 |  |  |  |  |  |  |
|  |  | 35-39 | 2.2 | 51.6 | 30.1 | 16.1 | 13 | 1,709 |  |  |  |  |  |  |
|  |  | 40-44 | 1.8 | 40.6 | 49.4 | b | 1 | 1,579 |  |  |  |  |  |  |
|  |  | 45-49 | 2.5 | 49.8 | 47.8 | b | 1 | 1,590 |  |  |  |  |  |  |
|  |  |  |  |  |  |  |  |  |  |  |  |  |  |  |
| South | Tamil Nadu | 15-19 | 90.1 | 0.7 | 6.6 | 2.6 | 39 | 3,979 |  |  |  |  |  |  |
|  |  | 20-24 | 55.5 | 15.1 | 18.2 | 11.2 | 152 | 4,409 |  |  |  |  |  |  |
|  |  | 25-29 | 12.7 | 49.9 | 29.9 | 7.4 | 113 | 4,708 |  |  |  |  |  |  |
|  |  | 30-34 | 4.1 | 54.8 | 41.1 | 0.0 | 31 | 4,130 |  |  |  |  |  |  |
|  |  | 35-39 | 3.1 | 42.9 | 53.9 | 0.0 | 5 | 4,239 |  |  |  |  |  |  |
|  |  | 40-44 | 2.6 | 35.6 | 49.1 | b | 1 | 3,523 |  |  |  |  |  |  |
|  |  | 45-49 | 2.4 | 33.6 | 64.0 | b | 0* | 3,832 |  |  |  |  |  |  |
|  |  |  |  |  |  |  |  |  |  |  |  |  |  |  |
| South | Telangana | 15-19 | 87.6 | 0.5 | 5.9 | 6.0 | 67 | 1,053 |  |  |  |  |  |  |
|  |  | 20-24 | 43.6 | 18.0 | 17.5 | 20.9 | 176 | 1,361 |  |  |  |  |  |  |
|  |  | 25-29 | 8.5 | 46.7 | 28.4 | 16.5 | 89 | 1,391 |  |  |  |  |  |  |
|  |  | 30-34 | 4.2 | 46.3 | 32.6 | 16.8 | 17 | 1,025 |  |  |  |  |  |  |
|  |  | 35-39 | 2.9 | 45.9 | 31.0 | b | 5 | 1,013 |  |  |  |  |  |  |
|  |  | 40-44 | 3.7 | 44.9 | 51.4 | b | 1 | 826 |  |  |  |  |  |  |
|  |  | 45-49 | 5.1 | 51.8 | 43.1 | b | 2 | 898 |  |  |  |  |  |  |
| § The proportionate reduction in fertility (from the Total Fecundity Rate to the actual Total Fertility Rate) that is attributable to each proximate determinant | | | | | | | | | | | | | | |
| * Values appear as 0 because they are less than 0.5 | | | | | | | | | | |  |  |  |  |
| a = cell count less than 50 cases (unweighted) | | | | | | | | | | |  |  |  |  |
| [ ] = cell count between 50 -100 cases (unweighted) | | | | | | | | | | |  |  |  |  |
| b = no. of women who have given birth in past 3 years less than 25 cases (unweighted) | | | | | | | | | | | | | |  |
| { } = no. of women who have given birth in past 3 years between 25 - 50 cases (unweighted) | | | | | | | | | | | | | |  |
| c = The values of the ASFR and the age-specific fecundity rate for this group are 0; the contributions of the proximate determinants cannot be defined. | | | | | | | | | | | | | | |
