# Supplementary material for: Key drivers of fertility levels and differentials in India, at the national, state and population subgroup levels, 2015–2016: An application of Bongaarts’ proximate determinants model
Source: PLoS One. 2022 Feb 7;17(2):e0263532. doi: 10.1371/journal.pone.0263532 (PMC8820640; doi:10.1371/journal.pone.0263532)
Supplement: S3 Table — (DOCX) [file pone.0263532.s003.docx]

**S3 Table: Estimates of the indices of four key proximate determinants of fertility by age-group: National and by state, India 2015-16**

|  |  |  | **Indices** | | | |
| --- | --- | --- | --- | --- | --- | --- |
| **Region** | **National/State** | **Age group** | **Marriage  (Cm)** | **Contra- ception  (Cc)** | **Induced  Abortion  (Ca)** | **Postpartum  Infecund- ability  (Ci)** |
| National | National | 15-19 | 0.15 | 0.94 | 0.83 | 0.89 |
|  |  | 20-24 | 0.65 | 0.84 | 0.81 | 0.82 |
|  |  | 25-29 | 0.89 | 0.62 | 0.73 | 0.78 |
|  |  | 30-34 | 0.94 | 0.39 | 0.63 | 0.73 |
|  |  | 35-39 | 0.93 | 0.27 | 0.47 | 0.70 |
|  |  | 40-44 | 0.90 | 0.18 | 0.26 | 0.66 |
|  |  | 45-49 | 0.87 | 0.10 | 0.18 | 0.62 |
|  |  |  |  |  |  |  |
| North | Haryana | 15-19 | 0.11 | 0.92 | 0.78 | 0.89 |
|  |  | 20-24 | 0.67 | 0.80 | 0.80 | 0.85 |
|  |  | 25-29 | 0.93 | 0.52 | 0.71 | 0.78 |
|  |  | 30-34 | 0.96 | 0.27 | 0.54 | 0.75 |
|  |  | 35-39 | 0.95 | 0.13 | 0.40 | 0.60 |
|  |  | 40-44 | 0.93 | 0.10 | 0.25 | 0.66 |
|  |  | 45-49 | 0.89 | 0.10 | 0.15 | 1.00 |
|  |  |  |  |  |  |  |
| North | Himachal Pradesh | 15-19 | 0.07 | 0.95 | 0.68 | 0.93 |
|  |  | 20-24 | 0.52 | 0.82 | 0.75 | 0.93 |
|  |  | 25-29 | 0.89 | 0.63 | 0.68 | 0.93 |
|  |  | 30-34 | 0.95 | 0.40 | 0.54 | 0.93 |
|  |  | 35-39 | 0.94 | 0.23 | 0.27 | 0.98 |
|  |  | 40-44 | 0.94 | 0.17 | 0.14 | 1.00 |
|  |  | 45-49 | 0.88 | 0.10 | 0.18 | 0.44 |
|  |  |  |  |  |  |  |
| North | Jammu & Kashmir | 15-19 | 0.04 | 0.95 | 0.62 | 0.89 |
|  |  | 20-24 | 0.32 | 0.86 | 0.71 | 0.82 |
|  |  | 25-29 | 0.69 | 0.69 | 0.75 | 0.75 |
|  |  | 30-34 | 0.89 | 0.45 | 0.73 | 0.75 |
|  |  | 35-39 | 0.95 | 0.27 | 0.57 | 0.70 |
|  |  | 40-44 | 0.95 | 0.14 | 0.30 | 0.78 |
|  |  | 45-49 | 0.92 | 0.10 | 0.24 | 0.78 |
|  |  |  |  |  |  |  |
| North | Punjab | 15-19 | 0.05 | 0.88 | 0.63 | 0.89 |
|  |  | 20-24 | 0.43 | 0.73 | 0.71 | 0.89 |
|  |  | 25-29 | 0.81 | 0.46 | 0.67 | 0.93 |
|  |  | 30-34 | 0.95 | 0.21 | 0.55 | 0.82 |
|  |  | 35-39 | 0.95 | 0.10 | 0.35 | 0.89 |
|  |  | 40-44 | 0.92 | 0.10 | 0.09 | 0.98 |
|  |  | 45-49 | 0.89 | 0.10 | 0.00 | 1.00 |
|  |  |  |  |  |  |  |
| North | Rajasthan | 15-19 | 0.15 | 0.93 | 0.79 | 0.93 |
|  |  | 20-24 | 0.72 | 0.83 | 0.81 | 0.89 |
|  |  | 25-29 | 0.94 | 0.56 | 0.71 | 0.89 |
|  |  | 30-34 | 0.97 | 0.27 | 0.59 | 0.85 |
|  |  | 35-39 | 0.96 | 0.15 | 0.44 | 0.78 |
|  |  | 40-44 | 0.94 | 0.10 | 0.29 | 0.82 |
|  |  | 45-49 | 0.90 | 0.10 | 0.26 | 0.73 |
|  |  |  |  |  |  |  |
| North | Uttarakhand | 15-19 | 0.06 | 0.96 | 0.66 | 0.93 |
|  |  | 20-24 | 0.55 | 0.86 | 0.79 | 0.85 |
|  |  | 25-29 | 0.86 | 0.63 | 0.73 | 0.78 |
|  |  | 30-34 | 0.95 | 0.41 | 0.63 | 0.75 |
|  |  | 35-39 | 0.94 | 0.24 | 0.38 | 0.85 |
|  |  | 40-44 | 0.91 | 0.18 | 0.18 | 0.70 |
|  |  | 45-49 | 0.86 | 0.10 | 0.21 | 0.89 |
|  |  |  |  |  |  |  |
| Central | Chhattisgarh | 15-19 | 0.08 | 0.96 | 0.74 | 0.89 |
|  |  | 20-24 | 0.63 | 0.89 | 0.79 | 0.85 |
|  |  | 25-29 | 0.87 | 0.63 | 0.70 | 0.78 |
|  |  | 30-34 | 0.92 | 0.33 | 0.62 | 0.70 |
|  |  | 35-39 | 0.91 | 0.18 | 0.45 | 0.66 |
|  |  | 40-44 | 0.89 | 0.10 | 0.25 | 0.58 |
|  |  | 45-49 | 0.85 | 0.10 | 0.08 | 0.46 |
|  |  |  |  |  |  |  |
| Central | Madhya Pradesh | 15-19 | 0.14 | 0.96 | 0.81 | 0.85 |
|  |  | 20-24 | 0.72 | 0.88 | 0.82 | 0.78 |
|  |  | 25-29 | 0.93 | 0.62 | 0.70 | 0.75 |
|  |  | 30-34 | 0.96 | 0.38 | 0.55 | 0.75 |
|  |  | 35-39 | 0.94 | 0.26 | 0.36 | 0.73 |
|  |  | 40-44 | 0.92 | 0.20 | 0.17 | 0.75 |
|  |  | 45-49 | 0.89 | 0.10 | 0.13 | 0.78 |
|  |  |  |  |  |  |  |
| Central | Uttar Pradesh | 15-19 | 0.08 | 0.95 | 0.66 | 0.93 |
|  |  | 20-24 | 0.60 | 0.87 | 0.78 | 0.85 |
|  |  | 25-29 | 0.91 | 0.71 | 0.75 | 0.82 |
|  |  | 30-34 | 0.96 | 0.49 | 0.67 | 0.78 |
|  |  | 35-39 | 0.95 | 0.36 | 0.57 | 0.75 |
|  |  | 40-44 | 0.93 | 0.30 | 0.37 | 0.66 |
|  |  | 45-49 | 0.91 | 0.28 | 0.31 | 0.52 |
|  |  |  |  |  |  |  |
| East | Bihar | 15-19 | 0.24 | 0.99 | 0.88 | 0.85 |
|  |  | 20-24 | 0.83 | 0.96 | 0.87 | 0.75 |
|  |  | 25-29 | 0.96 | 0.84 | 0.80 | 0.73 |
|  |  | 30-34 | 0.97 | 0.68 | 0.76 | 0.66 |
|  |  | 35-39 | 0.96 | 0.61 | 0.68 | 0.62 |
|  |  | 40-44 | 0.93 | 0.56 | 0.51 | 0.60 |
|  |  | 45-49 | 0.91 | 0.51 | 0.42 | 0.60 |
|  |  |  |  |  |  |  |
| East | Jharkhand | 15-19 | 0.23 | 0.97 | 0.87 | 0.85 |
|  |  | 20-24 | 0.73 | 0.92 | 0.83 | 0.75 |
|  |  | 25-29 | 0.90 | 0.71 | 0.75 | 0.66 |
|  |  | 30-34 | 0.94 | 0.50 | 0.66 | 0.63 |
|  |  | 35-39 | 0.93 | 0.38 | 0.49 | 0.68 |
|  |  | 40-44 | 0.90 | 0.33 | 0.33 | 0.73 |
|  |  | 45-49 | 0.85 | 0.19 | 0.27 | 0.44 |
|  |  |  |  |  |  |  |
| East | Odisha | 15-19 | 0.14 | 0.90 | 0.80 | 0.85 |
|  |  | 20-24 | 0.61 | 0.81 | 0.79 | 0.70 |
|  |  | 25-29 | 0.86 | 0.63 | 0.76 | 0.60 |
|  |  | 30-34 | 0.91 | 0.41 | 0.69 | 0.52 |
|  |  | 35-39 | 0.91 | 0.30 | 0.56 | 0.46 |
|  |  | 40-44 | 0.88 | 0.23 | 0.31 | 0.55 |
|  |  | 45-49 | 0.87 | 0.10 | 0.20 | 0.60 |
|  |  |  |  |  |  |  |
| East | West Bengal | 15-19 | 0.30 | 0.82 | 0.89 | 0.85 |
|  |  | 20-24 | 0.77 | 0.65 | 0.81 | 0.60 |
|  |  | 25-29 | 0.90 | 0.41 | 0.67 | 0.56 |
|  |  | 30-34 | 0.94 | 0.19 | 0.58 | 0.48 |
|  |  | 35-39 | 0.92 | 0.14 | 0.32 | 0.49 |
|  |  | 40-44 | 0.90 | 0.10 | 0.14 | 0.41 |
|  |  | 45-49 | 0.88 | 0.10 | 0.04 | 0.43 |
|  |  |  |  |  |  |  |
| Northeast | Arunachal Pradesh | 15-19 | 0.16 | 0.92 | 0.80 | 0.85 |
|  |  | 20-24 | 0.58 | 0.88 | 0.72 | 0.73 |
|  |  | 25-29 | 0.83 | 0.78 | 0.68 | 0.68 |
|  |  | 30-34 | 0.90 | 0.65 | 0.65 | 0.66 |
|  |  | 35-39 | 0.93 | 0.57 | 0.59 | 0.60 |
|  |  | 40-44 | 0.89 | 0.57 | 0.51 | 0.60 |
|  |  | 45-49 | 0.84 | 0.64 | 0.29 | 0.68 |
|  |  |  |  |  |  |  |
| Northeast | Assam | 15-19 | 0.22 | 0.87 | 0.84 | 0.82 |
|  |  | 20-24 | 0.67 | 0.74 | 0.75 | 0.68 |
|  |  | 25-29 | 0.84 | 0.56 | 0.69 | 0.66 |
|  |  | 30-34 | 0.90 | 0.42 | 0.66 | 0.58 |
|  |  | 35-39 | 0.90 | 0.37 | 0.53 | 0.56 |
|  |  | 40-44 | 0.86 | 0.40 | 0.26 | 0.75 |
|  |  | 45-49 | 0.80 | 0.57 | 0.19 | 0.60 |
|  |  |  |  |  |  |  |
| Northeast | Manipur | 15-19 | 0.10 | 0.96 | 0.75 | 0.89 |
|  |  | 20-24 | 0.48 | 0.89 | 0.72 | 0.82 |
|  |  | 25-29 | 0.70 | 0.84 | 0.72 | 0.70 |
|  |  | 30-34 | 0.81 | 0.76 | 0.74 | 0.73 |
|  |  | 35-39 | 0.84 | 0.71 | 0.68 | 0.73 |
|  |  | 40-44 | 0.84 | 0.74 | 0.56 | 0.47 |
|  |  | 45-49 | 0.82 | 0.78 | 0.06 | 1.00 |
|  |  |  |  |  |  |  |
| Northeast | Meghalaya | 15-19 | 0.09 | 0.96 | 0.78 | 0.85 |
|  |  | 20-24 | 0.45 | 0.92 | 0.74 | 0.75 |
|  |  | 25-29 | 0.74 | 0.82 | 0.74 | 0.73 |
|  |  | 30-34 | 0.85 | 0.73 | 0.74 | 0.75 |
|  |  | 35-39 | 0.86 | 0.65 | 0.76 | 0.73 |
|  |  | 40-44 | 0.80 | 0.62 | 0.60 | 0.78 |
|  |  | 45-49 | 0.77 | 0.76 | 0.57 | 0.46 |
|  |  |  |  |  |  |  |
| Northeast | Mizoram | 15-19 | 0.07 | 0.96 | 0.72 | 0.93 |
|  |  | 20-24 | 0.31 | 0.85 | 0.67 | 0.89 |
|  |  | 25-29 | 0.53 | 0.75 | 0.67 | 0.82 |
|  |  | 30-34 | 0.74 | 0.64 | 0.66 | 0.85 |
|  |  | 35-39 | 0.74 | 0.58 | 0.61 | 0.85 |
|  |  | 40-44 | 0.75 | 0.50 | 0.50 | 0.63 |
|  |  | 45-49 | 0.78 | 0.45 | 0.16 | 0.93 |
|  |  |  |  |  |  |  |
| Northeast | Nagaland | 15-19 | 0.07 | 0.99 | 0.74 | 0.89 |
|  |  | 20-24 | 0.42 | 0.92 | 0.73 | 0.82 |
|  |  | 25-29 | 0.64 | 0.84 | 0.68 | 0.93 |
|  |  | 30-34 | 0.79 | 0.70 | 0.67 | 0.98 |
|  |  | 35-39 | 0.85 | 0.63 | 0.65 | 0.93 |
|  |  | 40-44 | 0.86 | 0.57 | 0.43 | 1.00 |
|  |  | 45-49 | 0.85 | 0.62 | 0.35 | 1.00 |
|  |  |  |  |  |  |  |
| Northeast | Sikkim | 15-19 | a | a | a | a |
|  |  | 20-24 | 0.39 | 0.82 | 0.60 | 0.75 |
|  |  | 25-29 | 0.71 | 0.67 | 0.58 | 0.63 |
|  |  | 30-34 | 0.84 | 0.53 | 0.56 | 0.63 |
|  |  | 35-39 | 0.86 | 0.42 | 0.39 | 0.70 |
|  |  | 40-44 | 0.89 | 0.30 | 0.06 | 0.44 |
|  |  | 45-49 | 0.85 | 0.23 | 0.10 | 0.41 |
|  |  |  |  |  |  |  |
| Northeast | Tripura | 15-19 | 0.25 | 0.87 | 0.86 | 0.85 |
|  |  | 20-24 | 0.69 | 0.71 | 0.76 | 0.53 |
|  |  | 25-29 | 0.88 | 0.50 | 0.62 | 0.63 |
|  |  | 30-34 | 0.90 | 0.25 | 0.56 | 0.51 |
|  |  | 35-39 | 0.94 | 0.23 | 0.42 | 0.55 |
|  |  | 40-44 | 0.85 | 0.25 | 0.09 | 0.41 |
|  |  | 45-49 | 0.85 | 0.25 | 0.02 | 0.85 |
|  |  |  |  |  |  |  |
| West | Goa | 15-19 | a | a | a | a |
|  |  | 20-24 | [.28] | [.88] | [.73] | [.68] |
|  |  | 25-29 | 0.74 | 0.77 | 0.80 | 0.68 |
|  |  | 30-34 | 0.89 | 0.71 | 0.75 | 0.78 |
|  |  | 35-39 | 0.90 | 0.70 | 0.71 | 0.70 |
|  |  | 40-44 | 0.86 | 0.67 | 0.46 | 0.46 |
|  |  | 45-49 | 0.84 | 0.65 | 0.29 | 1.00 |
|  |  |  |  |  |  |  |
| West | Gujarat | 15-19 | 0.14 | 0.93 | 0.80 | 0.85 |
|  |  | 20-24 | 0.62 | 0.87 | 0.79 | 0.89 |
|  |  | 25-29 | 0.90 | 0.67 | 0.71 | 0.89 |
|  |  | 30-34 | 0.95 | 0.49 | 0.53 | 0.98 |
|  |  | 35-39 | 0.94 | 0.35 | 0.38 | 0.85 |
|  |  | 40-44 | 0.91 | 0.25 | 0.13 | 0.85 |
|  |  | 45-49 | 0.88 | 0.10 | 0.10 | 0.68 |
|  |  |  |  |  |  |  |
| West | Maharashtra | 15-19 | 0.17 | 0.95 | 0.88 | 0.89 |
|  |  | 20-24 | 0.64 | 0.81 | 0.84 | 0.85 |
|  |  | 25-29 | 0.88 | 0.52 | 0.72 | 0.82 |
|  |  | 30-34 | 0.92 | 0.25 | 0.54 | 0.78 |
|  |  | 35-39 | 0.90 | 0.11 | 0.41 | 0.58 |
|  |  | 40-44 | 0.88 | 0.10 | 0.12 | 0.63 |
|  |  | 45-49 | 0.85 | 0.10 | 0.00 | 0.38 |
|  |  |  |  |  |  |  |
| South | Andhra Pradesh | 15-19 | 0.23 | 0.98 | 0.91 | 0.89 |
|  |  | 20-24 | 0.73 | 0.80 | 0.84 | 0.89 |
|  |  | 25-29 | 0.90 | 0.41 | 0.65 | 0.89 |
|  |  | 30-34 | 0.91 | 0.16 | 0.43 | 0.78 |
|  |  | 35-39 | 0.88 | 0.10 | 0.18 | 0.89 |
|  |  | 40-44 | 0.86 | 0.10 | 0.10 | 0.83 |
|  |  | 45-49 | 0.83 | 0.10 | 0.00 | 0.66 |
|  |  |  |  |  |  |  |
| South | Karnataka | 15-19 | 0.16 | 0.98 | 0.86 | 0.93 |
|  |  | 20-24 | 0.64 | 0.88 | 0.84 | 0.82 |
|  |  | 25-29 | 0.86 | 0.65 | 0.74 | 0.78 |
|  |  | 30-34 | 0.91 | 0.40 | 0.54 | 0.82 |
|  |  | 35-39 | 0.89 | 0.25 | 0.29 | 0.98 |
|  |  | 40-44 | 0.85 | 0.17 | 0.08 | 0.75 |
|  |  | 45-49 | 0.80 | 0.10 | 0.03 | 1.00 |
|  |  |  |  |  |  |  |
| South | Kerala | 15-19 | [.06] | [.91] | [.74] | [.78] |
|  |  | 20-24 | 0.47 | 0.93 | 0.78 | 0.78 |
|  |  | 25-29 | 0.87 | 0.75 | 0.74 | 0.89 |
|  |  | 30-34 | 0.96 | 0.49 | 0.67 | 0.75 |
|  |  | 35-39 | 0.95 | 0.29 | 0.48 | 0.68 |
|  |  | 40-44 | 0.92 | 0.15 | 0.10 | 0.68 |
|  |  | 45-49 | 0.89 | 0.10 | 0.11 | 1.00 |
|  |  |  |  |  |  |  |
| South | Tamil Nadu | 15-19 | 0.08 | 0.98 | 0.83 | 0.93 |
|  |  | 20-24 | 0.56 | 0.85 | 0.83 | 0.89 |
|  |  | 25-29 | 0.88 | 0.61 | 0.75 | 0.93 |
|  |  | 30-34 | 0.93 | 0.39 | 0.50 | 1.00 |
|  |  | 35-39 | 0.91 | 0.28 | 0.20 | 1.00 |
|  |  | 40-44 | 0.89 | 0.19 | 0.10 | 0.55 |
|  |  | 45-49 | 0.85 | 0.10 | 0.01 | 1.00 |
|  |  |  |  |  |  |  |
| South | Telangana | 15-19 | 0.18 | 0.99 | 0.89 | 0.89 |
|  |  | 20-24 | 0.65 | 0.84 | 0.84 | 0.82 |
|  |  | 25-29 | 0.90 | 0.56 | 0.70 | 0.82 |
|  |  | 30-34 | 0.90 | 0.31 | 0.44 | 0.66 |
|  |  | 35-39 | 0.90 | 0.18 | 0.31 | 0.47 |
|  |  | 40-44 | 0.83 | 0.10 | 0.07 | 1.00 |
|  |  | 45-49 | 0.80 | 0.10 | 0.15 | 1.00 |

a = cell count less than 50 cases (unweighted)

[ ] = cell count between 50 -100 cases (unweighted)
