# Supplementary material for: Key drivers of fertility levels and differentials in India, at the national, state and population subgroup levels, 2015–2016: An application of Bongaarts’ proximate determinants model
Source: PLoS One. 2022 Feb 7;17(2):e0263532. doi: 10.1371/journal.pone.0263532 (PMC8820640; doi:10.1371/journal.pone.0263532)
Supplement: S4 Table — (DOCX) [file pone.0263532.s004.docx]

**S4 Table: Estimates of the indices of four key proximate determinants of fertility by sub-group: National and by state, India 2015-16**

|  |  | **Indices** | | | |
| --- | --- | --- | --- | --- | --- |
| **Sub-group** | | **Marriage  (Cm)** | **Contra- ception  (Cc)** | **Induced  Abortion  (Ca)** | **Postpartum  Infecund- ability  (Ci)** |
| **India** |  | 0.59 | 0.70 | 0.71 | 0.78 |
| Residence | |  |  |  |  |
|  | Urban | 0.48 | 0.67 | 0.66 | 0.85 |
|  | Rural | 0.59 | 0.69 | 0.72 | 0.78 |
| Education | |  |  |  |  |
|  | <5 years | 0.75 | 0.69 | 0.81 | 0.75 |
|  | 5-9 years | 0.56 | 0.67 | 0.72 | 0.78 |
|  | 10 years plus | 0.48 | 0.70 | 0.61 | 0.85 |
| Wealth status | |  |  |  |  |
|  | Low | 0.61 | 0.72 | 0.74 | 0.75 |
|  | Middle | 0.55 | 0.66 | 0.71 | 0.82 |
|  | High | 0.48 | 0.67 | 0.65 | 0.89 |
| Caste |  |  |  |  |  |
|  | Scheduled caste/tribe | 0.57 | 0.68 | 0.74 | 0.75 |
|  | Other Backward Classes | 0.55 | 0.70 | 0.70 | 0.82 |
|  | Others | 0.52 | 0.66 | 0.68 | 0.82 |
| **North** |  |  |  |  |  |
| **Haryana** |  | 0.57 | 0.65 | 0.67 | 0.82 |
| Residence | |  |  |  |  |
|  | Urban | 0.54 | 0.68 | 0.62 | 0.82 |
|  | Rural | 0.57 | 0.61 | 0.73 | 0.82 |
| Education | |  |  |  |  |
|  | <5 years | 0.79 | 0.68 | 0.78 | 0.78 |
|  | 5-9 years | 0.55 | 0.62 | 0.73 | 0.78 |
|  | 10 years plus | 0.51 | 0.64 | 0.60 | 0.85 |
| Wealth status | |  |  |  |  |
|  | Low | 0.58 | 0.72 | 0.70 | 0.82 |
|  | Middle | 0.56 | 0.65 | 0.76 | 0.78 |
|  | High | 0.55 | 0.62 | 0.74 | 0.85 |
| Caste |  |  |  |  |  |
|  | Scheduled caste/tribe | 0.56 | 0.64 | 0.71 | 0.78 |
|  | Other Backward Classes | 0.57 | 0.62 | 0.68 | 0.82 |
|  | Others | 0.51 | 0.65 | 0.63 | 0.85 |
|  |  |  |  |  |  |
| **Himachal Pradesh** | | 0.50 | 0.69 | 0.62 | 0.93 |
| Residence | |  |  |  |  |
|  | Urban | a | a | a | a |
|  | Rural | 0.47 | 0.68 | 0.65 | 0.93 |
| Education | |  |  |  |  |
|  | <5 years | a | a | a | a |
|  | 5-9 years | 0.49 | 0.65 | 0.71 | 0.93 |
|  | 10 years plus | 0.47 | 0.70 | 0.51 | 0.93 |
| Wealth status | |  |  |  |  |
|  | Low | [.48] | [.58] | [.70] | [1.0] |
|  | Middle | 0.45 | 0.66 | 0.63 | 0.93 |
|  | High | 0.47 | 0.71 | 0.57 | 0.93 |
| Caste |  |  |  |  |  |
|  | Scheduled caste/tribe | 0.50 | 0.65 | 0.66 | 0.93 |
|  | Other Backward Classes | [.42] | [.70] | [.61] | [.82] |
|  | Others | 0.45 | 0.69 | 0.60 | 0.98 |
|  |  |  |  |  |  |
| **Jammu & Kashmir** | | 0.42 | 0.69 | 0.67 | 0.78 |
| Residence | |  |  |  |  |
|  | Urban | 0.31 | 0.63 | 0.65 | 0.89 |
|  | Rural | 0.42 | 0.70 | 0.66 | 0.75 |
| Education | |  |  |  |  |
|  | <5 years | 0.63 | 0.68 | 0.79 | 0.75 |
|  | 5-9 years | 0.40 | 0.67 | 0.67 | 0.78 |
|  | 10 years plus | 0.33 | 0.68 | 0.62 | 0.85 |
| Wealth status | |  |  |  |  |
|  | Low | 0.50 | 0.71 | 0.72 | 0.75 |
|  | Middle | 0.37 | 0.68 | 0.69 | 0.78 |
|  | High | 0.35 | 0.68 | 0.66 | 0.82 |
| Caste |  |  |  |  |  |
|  | Scheduled caste/tribe | 0.51 | 0.72 | 0.68 | 0.78 |
|  | Other Backward Classes | [.42] | [.69] | [.68] | [.89] |
|  | Others | 0.36 | 0.67 | 0.70 | 0.78 |
|  |  |  |  |  |  |
| **Punjab** |  | 0.44 | 0.57 | 0.59 | 0.89 |
| Residence | |  |  |  |  |
|  | Urban | 0.42 | 0.56 | 0.63 | 0.89 |
|  | Rural | 0.42 | 0.56 | 0.58 | 0.89 |
| Education | |  |  |  |  |
|  | <5 years | 0.68 | 0.52 | 0.80 | 0.82 |
|  | 5-9 years | 0.47 | 0.56 | 0.64 | 0.89 |
|  | 10 years plus | 0.39 | 0.57 | 0.51 | 0.93 |
| Wealth status | |  |  |  |  |
|  | Low | [.50] | [.51] | [.66] | [.89] |
|  | Middle | 0.45 | 0.56 | 0.68 | 0.82 |
|  | High | 0.41 | 0.56 | 0.58 | 0.93 |
| Caste |  |  |  |  |  |
|  | Scheduled caste/tribe | 0.43 | 0.56 | 0.62 | 0.89 |
|  | Other Backward Classes | 0.45 | 0.56 | 0.64 | 0.89 |
|  | Others | 0.40 | 0.55 | 0.56 | 0.89 |
|  |  |  |  |  |  |
| **Rajasthan** | | 0.63 | 0.65 | 0.68 | 0.89 |
| Residence | |  |  |  |  |
|  | Urban | 0.51 | 0.62 | 0.63 | 0.93 |
|  | Rural | 0.62 | 0.64 | 0.70 | 0.89 |
| Education | |  |  |  |  |
|  | <5 years | 0.76 | 0.63 | 0.80 | 0.85 |
|  | 5-9 years | 0.59 | 0.66 | 0.66 | 0.89 |
|  | 10 years plus | 0.50 | 0.64 | 0.58 | 0.93 |
| Wealth status | |  |  |  |  |
|  | Low | 0.65 | 0.65 | 0.74 | 0.85 |
|  | Middle | 0.59 | 0.64 | 0.67 | 0.89 |
|  | High | 0.54 | 0.63 | 0.63 | 0.93 |
| Caste |  |  |  |  |  |
|  | Scheduled caste/tribe | 0.62 | 0.65 | 0.71 | 0.85 |
|  | Other Backward Classes | 0.60 | 0.62 | 0.68 | 0.89 |
|  | Others | 0.51 | 0.63 | 0.65 | 0.89 |
|  |  |  |  |  |  |
| **Uttarakhand** | | 0.51 | 0.70 | 0.67 | 0.82 |
| Residence | |  |  |  |  |
|  | Urban | 0.44 | 0.69 | 0.65 | 0.89 |
|  | Rural | 0.49 | 0.70 | 0.69 | 0.75 |
| Education | |  |  |  |  |
|  | <5 years | 0.69 | 0.71 | 0.78 | 0.78 |
|  | 5-9 years | 0.50 | 0.71 | 0.71 | 0.82 |
|  | 10 years plus | 0.42 | 0.69 | 0.61 | 0.82 |
| Wealth status | |  |  |  |  |
|  | Low | 0.49 | 0.69 | 0.73 | 0.78 |
|  | Middle | 0.50 | 0.71 | 0.69 | 0.75 |
|  | High | 0.45 | 0.68 | 0.64 | 0.89 |
| Caste |  |  |  |  |  |
|  | Scheduled caste/tribe | 0.46 | 0.71 | 0.70 | 0.75 |
|  | Other Backward Classes | 0.51 | 0.69 | 0.68 | 0.93 |
|  | Others | 0.46 | 0.70 | 0.67 | 0.75 |
| **Central** |  |  |  |  |  |
| **Chhattisgarh** | | 0.55 | 0.71 | 0.66 | 0.82 |
| Residence | |  |  |  |  |
|  | Urban | 0.46 | 0.68 | 0.60 | 0.89 |
|  | Rural | 0.53 | 0.71 | 0.68 | 0.82 |
| Education | |  |  |  |  |
|  | <5 years | 0.74 | 0.71 | 0.79 | 0.78 |
|  | 5-9 years | 0.51 | 0.69 | 0.69 | 0.82 |
|  | 10 years plus | 0.45 | 0.72 | 0.55 | 0.89 |
| Wealth status | |  |  |  |  |
|  | Low | 0.53 | 0.72 | 0.69 | 0.78 |
|  | Middle | 0.50 | 0.68 | 0.67 | 0.85 |
|  | High | 0.47 | 0.70 | 0.60 | 0.89 |
| Caste |  |  |  |  |  |
|  | Scheduled caste/tribe | 0.54 | 0.72 | 0.67 | 0.82 |
|  | Other Backward Classes | 0.49 | 0.69 | 0.69 | 0.82 |
|  | Others | 0.44 | 0.70 | 0.61 | 0.85 |
|  |  |  |  |  |  |
| **Madhya Pradesh** | | 0.60 | 0.73 | 0.68 | 0.78 |
| Residence | |  |  |  |  |
|  | Urban | 0.49 | 0.72 | 0.61 | 0.85 |
|  | Rural | 0.60 | 0.71 | 0.72 | 0.75 |
| Education | |  |  |  |  |
|  | <5 years | 0.80 | 0.68 | 0.84 | 0.75 |
|  | 5-9 years | 0.56 | 0.72 | 0.67 | 0.78 |
|  | 10 years plus | 0.47 | 0.74 | 0.54 | 0.85 |
| Wealth status | |  |  |  |  |
|  | Low | 0.62 | 0.71 | 0.74 | 0.75 |
|  | Middle | 0.55 | 0.70 | 0.66 | 0.82 |
|  | High | 0.49 | 0.72 | 0.59 | 0.85 |
| Caste |  |  |  |  |  |
|  | Scheduled caste/tribe | 0.59 | 0.71 | 0.72 | 0.78 |
|  | Other Backward Classes | 0.57 | 0.71 | 0.68 | 0.78 |
|  | Others | 0.50 | 0.72 | 0.61 | 0.82 |
|  |  |  |  |  |  |
| **Uttar Pradesh** | | 0.59 | 0.72 | 0.69 | 0.82 |
| Residence | |  |  |  |  |
|  | Urban | 0.45 | 0.66 | 0.66 | 0.89 |
|  | Rural | 0.57 | 0.74 | 0.67 | 0.82 |
| Education | |  |  |  |  |
|  | <5 years | 0.68 | 0.74 | 0.77 | 0.78 |
|  | 5-9 years | 0.53 | 0.72 | 0.68 | 0.85 |
|  | 10 years plus | 0.47 | 0.72 | 0.60 | 0.85 |
| Wealth status | |  |  |  |  |
|  | Low | 0.58 | 0.77 | 0.70 | 0.78 |
|  | Middle | 0.54 | 0.71 | 0.67 | 0.85 |
|  | High | 0.47 | 0.67 | 0.65 | 0.93 |
| Caste |  |  |  |  |  |
|  | Scheduled caste/tribe | 0.58 | 0.74 | 0.72 | 0.78 |
|  | Other Backward Classes | 0.54 | 0.72 | 0.68 | 0.85 |
|  | Others | 0.48 | 0.70 | 0.65 | 0.89 |
| **East** |  |  |  |  |  |
| **Bihar** |  | 0.72 | 0.86 | 0.79 | 0.73 |
| Residence | |  |  |  |  |
|  | Urban | 0.53 | 0.81 | 0.73 | 0.78 |
|  | Rural | 0.68 | 0.86 | 0.80 | 0.73 |
| Education | |  |  |  |  |
|  | <5 years | 0.82 | 0.86 | 0.85 | 0.73 |
|  | 5-9 years | 0.57 | 0.85 | 0.75 | 0.75 |
|  | 10 years plus | 0.54 | 0.85 | 0.70 | 0.75 |
| Wealth status | |  |  |  |  |
|  | Low | 0.71 | 0.87 | 0.81 | 0.73 |
|  | Middle | 0.58 | 0.84 | 0.74 | 0.75 |
|  | High | 0.48 | 0.83 | 0.68 | 0.82 |
| Caste |  |  |  |  |  |
|  | Scheduled caste/tribe | 0.72 | 0.86 | 0.82 | 0.73 |
|  | Other Backward Classes | 0.67 | 0.85 | 0.80 | 0.73 |
|  | Others | 0.57 | 0.86 | 0.75 | 0.75 |
|  |  |  |  |  |  |
| **Jharkhand** | | 0.65 | 0.78 | 0.73 | 0.73 |
| Residence | |  |  |  |  |
|  | Urban | 0.45 | 0.77 | 0.68 | 0.73 |
|  | Rural | 0.66 | 0.77 | 0.75 | 0.70 |
| Education | |  |  |  |  |
|  | <5 years | 0.79 | 0.77 | 0.82 | 0.70 |
|  | 5-9 years | 0.58 | 0.76 | 0.74 | 0.70 |
|  | 10 years plus | 0.52 | 0.80 | 0.63 | 0.75 |
| Wealth status | |  |  |  |  |
|  | Low | 0.65 | 0.79 | 0.75 | 0.70 |
|  | Middle | 0.57 | 0.72 | 0.71 | 0.75 |
|  | High | 0.45 | 0.78 | 0.63 | 0.78 |
| Caste |  |  |  |  |  |
|  | Scheduled caste/tribe | 0.62 | 0.80 | 0.73 | 0.70 |
|  | Other Backward Classes | 0.61 | 0.74 | 0.75 | 0.73 |
|  | Others | 0.50 | 0.78 | 0.69 | 0.78 |
|  |  |  |  |  |  |
| **Odisha** |  | 0.57 | 0.70 | 0.72 | 0.62 |
| Residence | |  |  |  |  |
|  | Urban | 0.49 | 0.66 | 0.68 | 0.63 |
|  | Rural | 0.54 | 0.68 | 0.73 | 0.62 |
| Education | |  |  |  |  |
|  | <5 years | 0.70 | 0.67 | 0.82 | 0.62 |
|  | 5-9 years | 0.57 | 0.69 | 0.72 | 0.63 |
|  | 10 years plus | 0.44 | 0.69 | 0.63 | 0.63 |
| Wealth status | |  |  |  |  |
|  | Low | 0.54 | 0.69 | 0.74 | 0.63 |
|  | Middle | 0.55 | 0.68 | 0.72 | 0.60 |
|  | High | 0.48 | 0.66 | 0.66 | 0.66 |
| Caste |  |  |  |  |  |
|  | Scheduled caste/tribe | 0.57 | 0.68 | 0.75 | 0.63 |
|  | Other Backward Classes | 0.52 | 0.67 | 0.73 | 0.58 |
|  | Others | 0.47 | 0.71 | 0.67 | 0.63 |
|  |  |  |  |  |  |
| **West Bengal** | | 0.67 | 0.59 | 0.69 | 0.62 |
| Residence | |  |  |  |  |
|  | Urban | 0.55 | 0.57 | 0.62 | 0.70 |
|  | Rural | 0.66 | 0.55 | 0.73 | 0.60 |
| Education | |  |  |  |  |
|  | <5 years | 0.80 | 0.56 | 0.78 | 0.60 |
|  | 5-9 years | 0.66 | 0.55 | 0.69 | 0.62 |
|  | 10 years plus | 0.54 | 0.57 | 0.63 | 0.63 |
| Wealth status | |  |  |  |  |
|  | Low | 0.67 | 0.57 | 0.71 | 0.62 |
|  | Middle | 0.63 | 0.55 | 0.69 | 0.63 |
|  | High | 0.52 | 0.55 | 0.63 | 0.60 |
| Caste |  |  |  |  |  |
|  | Scheduled caste/tribe | 0.65 | 0.55 | 0.75 | 0.56 |
|  | Other Backward Classes | 0.56 | 0.52 | 0.65 | 0.66 |
|  | Others | 0.63 | 0.57 | 0.67 | 0.62 |
| **Northeast** | |  |  |  |  |
| **Arunachal Pradesh** | | 0.59 | 0.80 | 0.67 | 0.68 |
| Residence | |  |  |  |  |
|  | Urban | 0.50 | 0.83 | 0.62 | 0.66 |
|  | Rural | 0.57 | 0.79 | 0.71 | 0.68 |
| Education | |  |  |  |  |
|  | <5 years | 0.74 | 0.80 | 0.79 | 0.68 |
|  | 5-9 years | 0.54 | 0.76 | 0.68 | 0.68 |
|  | 10 years plus | 0.48 | 0.84 | 0.57 | 0.68 |
| Wealth status | |  |  |  |  |
|  | Low | 0.61 | 0.78 | 0.75 | 0.70 |
|  | Middle | 0.55 | 0.79 | 0.65 | 0.68 |
|  | High | 0.47 | 0.84 | 0.60 | 0.63 |
| Caste |  |  |  |  |  |
|  | Scheduled caste/tribe | 0.54 | 0.82 | 0.64 | 0.66 |
|  | Other Backward Classes | [.56] | [.72] | [.68] | [.70] |
|  | Others | 0.57 | 0.71 | 0.70 | 0.75 |
|  |  |  |  |  |  |
| **Assam** |  | 0.63 | 0.67 | 0.67 | 0.68 |
| Residence | |  |  |  |  |
|  | Urban | 0.49 | 0.61 | 0.56 | 0.75 |
|  | Rural | 0.61 | 0.66 | 0.67 | 0.68 |
| Education | |  |  |  |  |
|  | <5 years | 0.75 | 0.66 | 0.74 | 0.66 |
|  | 5-9 years | 0.60 | 0.65 | 0.66 | 0.68 |
|  | 10 years plus | 0.50 | 0.64 | 0.62 | 0.70 |
| Wealth status | |  |  |  |  |
|  | Low | 0.64 | 0.67 | 0.69 | 0.68 |
|  | Middle | 0.56 | 0.64 | 0.64 | 0.66 |
|  | High | 0.47 | 0.62 | 0.59 | 0.70 |
| Caste |  |  |  |  |  |
|  | Scheduled caste/tribe | 0.57 | 0.65 | 0.67 | 0.66 |
|  | Other Backward Classes | 0.54 | 0.65 | 0.64 | 0.68 |
|  | Others | 0.64 | 0.66 | 0.68 | 0.70 |
|  |  |  |  |  |  |
| **Manipur** |  | 0.52 | 0.85 | 0.69 | 0.75 |
| Residence | |  |  |  |  |
|  | Urban | 0.44 | 0.84 | 0.69 | 0.66 |
|  | Rural | 0.52 | 0.85 | 0.71 | 0.78 |
| Education | |  |  |  |  |
|  | <5 years | 0.61 | 0.86 | 0.75 | 0.82 |
|  | 5-9 years | 0.51 | 0.84 | 0.70 | 0.75 |
|  | 10 years plus | 0.45 | 0.85 | 0.69 | 0.70 |
| Wealth status | |  |  |  |  |
|  | Low | 0.50 | 0.87 | 0.71 | 0.82 |
|  | Middle | 0.49 | 0.83 | 0.69 | 0.73 |
|  | High | 0.47 | 0.86 | 0.72 | 0.66 |
| Caste |  |  |  |  |  |
|  | Scheduled caste/tribe | 0.50 | 0.87 | 0.73 | 0.82 |
|  | Other Backward Classes | 0.41 | 0.87 | 0.69 | 0.62 |
|  | Others | 0.51 | 0.83 | 0.71 | 0.66 |
|  |  |  |  |  |  |
| **Meghalaya** | | 0.54 | 0.83 | 0.72 | 0.75 |
| Residence | |  |  |  |  |
|  | Urban | 0.34 | 0.80 | 0.58 | 0.82 |
|  | Rural | 0.54 | 0.85 | 0.76 | 0.73 |
| Education | |  |  |  |  |
|  | <5 years | 0.67 | 0.84 | 0.80 | 0.75 |
|  | 5-9 years | 0.52 | 0.82 | 0.73 | 0.75 |
|  | 10 years plus | 0.38 | 0.84 | 0.67 | 0.66 |
| Wealth status | |  |  |  |  |
|  | Low | 0.63 | 0.86 | 0.77 | 0.75 |
|  | Middle | 0.46 | 0.83 | 0.70 | 0.75 |
|  | High | [.32] | [.79] | [.65] | [.66] |
| Caste |  |  |  |  |  |
|  | Scheduled caste/tribe | 0.49 | 0.85 | 0.71 | 0.75 |
|  | Other Backward Classes | a | a | a | a |
|  | Others | [.60] | [.70] | [.75] | [.73] |
|  |  |  |  |  |  |
| **Mizoram** |  | 0.40 | 0.78 | 0.62 | 0.89 |
| Residence | |  |  |  |  |
|  | Urban | 0.31 | 0.73 | 0.61 | 0.89 |
|  | Rural | 0.45 | 0.80 | 0.67 | 0.85 |
| Education | |  |  |  |  |
|  | <5 years | 0.60 | 0.84 | 0.74 | 0.85 |
|  | 5-9 years | 0.39 | 0.73 | 0.65 | 0.89 |
|  | 10 years plus | 0.29 | 0.77 | 0.58 | 0.85 |
| Wealth status | |  |  |  |  |
|  | Low | 0.60 | 0.86 | 0.72 | 0.82 |
|  | Middle | 0.41 | 0.74 | 0.66 | 0.93 |
|  | High | 0.29 | 0.74 | 0.62 | 0.85 |
| Caste |  |  |  |  |  |
|  | Scheduled caste/tribe | 0.37 | 0.76 | 0.66 | 0.89 |
|  | Other Backward Classes | a | a | a | a |
|  | Others | a | a | a | a |
|  |  |  |  |  |  |
| **Nagaland** |  | 0.48 | 0.82 | 0.65 | 0.93 |
| Residence | |  |  |  |  |
|  | Urban | 0.34 | 0.78 | 0.55 | 0.98 |
|  | Rural | 0.51 | 0.84 | 0.72 | 0.89 |
| Education | |  |  |  |  |
|  | <5 years | 0.69 | 0.83 | 0.77 | 0.85 |
|  | 5-9 years | 0.46 | 0.82 | 0.68 | 0.93 |
|  | 10 years plus | 0.30 | 0.81 | 0.57 | 0.93 |
| Wealth status | |  |  |  |  |
|  | Low | 0.61 | 0.85 | 0.75 | 0.89 |
|  | Middle | 0.40 | 0.80 | 0.63 | 0.93 |
|  | High | [.31] | [.79] | [.53] | [.98] |
| Caste |  |  |  |  |  |
|  | Scheduled caste/tribe | 0.43 | 0.83 | 0.65 | 0.93 |
|  | Other Backward Classes | a | a | a | a |
|  | Others | a | a | a | a |
|  |  |  |  |  |  |
| **Sikkim** |  | 0.44 | 0.73 | 0.53 | 0.68 |
| Residence | |  |  |  |  |
|  | Urban | 0.43 | 0.79 | 0.52 | 0.63 |
|  | Rural | 0.39 | 0.67 | 0.55 | 0.73 |
| Education | |  |  |  |  |
|  | <5 years | [.64] | [.65] | [.71] | [.66] |
|  | 5-9 years | 0.46 | 0.72 | 0.57 | 0.66 |
|  | 10 years plus | 0.33 | 0.77 | 0.44 | 0.75 |
| Wealth status | |  |  |  |  |
|  | Low | a | a | a | a |
|  | Middle | 0.40 | 0.68 | 0.54 | 0.73 |
|  | High | [.40] | [.77] | [.46] | [.68] |
| Caste |  |  |  |  |  |
|  | Scheduled caste/tribe | 0.38 | 0.69 | 0.51 | 0.70 |
|  | Other Backward Classes | 0.41 | 0.68 | 0.53 | 0.73 |
|  | Others | [.42] | [.79] | [.56] | [.68] |
|  |  |  |  |  |  |
| **Tripura** |  | 0.63 | 0.64 | 0.64 | 0.62 |
| Residence | |  |  |  |  |
|  | Urban | 0.55 | 0.58 | 0.62 | 0.55 |
|  | Rural | 0.60 | 0.61 | 0.66 | 0.63 |
| Education | |  |  |  |  |
|  | <5 years | 0.80 | 0.61 | 0.73 | 0.62 |
|  | 5-9 years | 0.60 | 0.61 | 0.64 | 0.66 |
|  | 10 years plus | 0.51 | 0.58 | 0.63 | 0.53 |
| Wealth status | |  |  |  |  |
|  | Low | 0.57 | 0.63 | 0.68 | 0.62 |
|  | Middle | 0.63 | 0.56 | 0.70 | 0.55 |
|  | High | [.52] | [.60] | [.47] | [.78] |
| Caste |  |  |  |  |  |
|  | Scheduled caste/tribe | 0.58 | 0.60 | 0.67 | 0.62 |
|  | Other Backward Classes | 0.59 | 0.61 | 0.69 | 0.55 |
|  | Others | 0.61 | 0.60 | 0.59 | 0.63 |
| **West** |  |  |  |  |  |
| **Goa** |  | 0.45 | 0.82 | 0.72 | 0.73 |
| Residence | |  |  |  |  |
|  | Urban | a | a | a | a |
|  | Rural | a | a | a | a |
| Education | |  |  |  |  |
|  | <5 years | a | a | a | a |
|  | 5-9 years | a | a | a | a |
|  | 10 years plus | a | a | a | a |
| Wealth status | |  |  |  |  |
|  | Low | a | a | a | a |
|  | Middle | a | a | a | a |
|  | High | a | a | a | a |
| Caste |  |  |  |  |  |
|  | Scheduled caste/tribe | a | a | a | a |
|  | Other Backward Classes | a | a | a | a |
|  | Others | a | a | a | a |
|  |  |  |  |  |  |
| **Gujarat** |  | 0.57 | 0.74 | 0.66 | 0.89 |
| Residence | |  |  |  |  |
|  | Urban | 0.52 | 0.72 | 0.60 | 0.98 |
|  | Rural | 0.56 | 0.73 | 0.71 | 0.85 |
| Education | |  |  |  |  |
|  | <5 years | 0.75 | 0.72 | 0.82 | 0.78 |
|  | 5-9 years | 0.58 | 0.72 | 0.67 | 0.89 |
|  | 10 years plus | 0.46 | 0.73 | 0.51 | 1.00 |
| Wealth status | |  |  |  |  |
|  | Low | 0.61 | 0.76 | 0.75 | 0.78 |
|  | Middle | 0.55 | 0.73 | 0.70 | 0.85 |
|  | High | 0.51 | 0.72 | 0.57 | 1.00 |
| Caste |  |  |  |  |  |
|  | Scheduled caste/tribe | 0.55 | 0.71 | 0.71 | 0.82 |
|  | Other Backward Classes | 0.56 | 0.73 | 0.66 | 0.89 |
|  | Others | 0.48 | 0.73 | 0.59 | 1.00 |
|  |  |  |  |  |  |
| **Maharashtra** | | 0.55 | 0.68 | 0.72 | 0.82 |
| Residence | |  |  |  |  |
|  | Urban | 0.47 | 0.65 | 0.67 | 0.85 |
|  | Rural | 0.58 | 0.65 | 0.77 | 0.82 |
| Education | |  |  |  |  |
|  | <5 years | 0.74 | 0.62 | 0.88 | 0.73 |
|  | 5-9 years | 0.60 | 0.64 | 0.77 | 0.78 |
|  | 10 years plus | 0.47 | 0.67 | 0.60 | 0.89 |
| Wealth status | |  |  |  |  |
|  | Low | 0.56 | 0.64 | 0.79 | 0.75 |
|  | Middle | 0.56 | 0.67 | 0.73 | 0.78 |
|  | High | 0.47 | 0.64 | 0.67 | 0.89 |
| Caste |  |  |  |  |  |
|  | Scheduled caste/tribe | 0.56 | 0.66 | 0.74 | 0.78 |
|  | Other Backward Classes | 0.48 | 0.65 | 0.72 | 0.85 |
|  | Others | 0.52 | 0.64 | 0.71 | 0.85 |
| **South** |  |  |  |  |  |
| **Andhra Pradesh** | | 0.59 | 0.69 | 0.70 | 0.89 |
| Residence | |  |  |  |  |
|  | Urban | 0.51 | 0.63 | 0.61 | 0.93 |
|  | Rural | 0.62 | 0.63 | 0.77 | 0.85 |
| Education | |  |  |  |  |
|  | <5 years | 0.86 | 0.56 | 0.89 | 0.82 |
|  | 5-9 years | 0.71 | 0.60 | 0.77 | 0.89 |
|  | 10 years plus | 0.51 | 0.69 | 0.57 | 0.89 |
| Wealth status | |  |  |  |  |
|  | Low | 0.66 | 0.65 | 0.78 | 0.82 |
|  | Middle | 0.62 | 0.61 | 0.76 | 0.85 |
|  | High | 0.50 | 0.67 | 0.59 | 0.93 |
| Caste |  |  |  |  |  |
|  | Scheduled caste/tribe | 0.60 | 0.66 | 0.75 | 0.78 |
|  | Other Backward Classes | 0.60 | 0.61 | 0.72 | 0.89 |
|  | Others | 0.51 | 0.66 | 0.63 | 1.00 |
|  |  |  |  |  |  |
| **Karnataka** | | 0.55 | 0.75 | 0.71 | 0.82 |
| Residence | |  |  |  |  |
|  | Urban | 0.49 | 0.75 | 0.68 | 0.78 |
|  | Rural | 0.56 | 0.71 | 0.77 | 0.82 |
| Education | |  |  |  |  |
|  | <5 years | 0.76 | 0.68 | 0.89 | 0.73 |
|  | 5-9 years | 0.59 | 0.70 | 0.75 | 0.82 |
|  | 10 years plus | 0.50 | 0.77 | 0.62 | 0.85 |
| Wealth status | |  |  |  |  |
|  | Low | 0.56 | 0.69 | 0.79 | 0.82 |
|  | Middle | 0.55 | 0.71 | 0.73 | 0.82 |
|  | High | 0.49 | 0.77 | 0.65 | 0.82 |
| Caste |  |  |  |  |  |
|  | Scheduled caste/tribe | 0.54 | 0.72 | 0.74 | 0.82 |
|  | Other Backward Classes | 0.52 | 0.72 | 0.71 | 0.85 |
|  | Others | 0.52 | 0.75 | 0.72 | 0.75 |
|  |  |  |  |  |  |
| **Kerala** |  | 0.49 | 0.74 | 0.67 | 0.85 |
| Residence | |  |  |  |  |
|  | Urban | 0.46 | 0.75 | 0.66 | 0.89 |
|  | Rural | 0.44 | 0.74 | 0.71 | 0.75 |
| Education | |  |  |  |  |
|  | <5 years | a | a | a | a |
|  | 5-9 years | [.36] | [.73] | [.75] | [.75] |
|  | 10 years plus | 0.47 | 0.77 | 0.56 | 0.85 |
| Wealth status | |  |  |  |  |
|  | Low | a | a | a | a |
|  | Middle | 0.44 | 0.72 | 0.71 | 0.75 |
|  | High | 0.46 | 0.77 | 0.63 | 0.85 |
| Caste |  |  |  |  |  |
|  | Scheduled caste/tribe | 0.45 | 0.71 | 0.79 | 0.55 |
|  | Other Backward Classes | 0.47 | 0.73 | 0.68 | 0.82 |
|  | Others | 0.42 | 0.77 | 0.64 | 0.89 |
|  |  |  |  |  |  |
| **Tamil Nadu** | | 0.50 | 0.73 | 0.69 | 0.93 |
| Residence | |  |  |  |  |
|  | Urban | 0.45 | 0.71 | 0.66 | 0.98 |
|  | Rural | 0.50 | 0.70 | 0.74 | 0.89 |
| Education | |  |  |  |  |
|  | <5 years | 0.72 | 0.64 | 0.90 | 0.78 |
|  | 5-9 years | 0.59 | 0.69 | 0.77 | 0.89 |
|  | 10 years plus | 0.46 | 0.73 | 0.60 | 0.93 |
| Wealth status | |  |  |  |  |
|  | Low | 0.47 | 0.71 | 0.77 | 0.85 |
|  | Middle | 0.49 | 0.69 | 0.72 | 0.89 |
|  | High | 0.46 | 0.72 | 0.65 | 0.98 |
| Caste |  |  |  |  |  |
|  | Scheduled caste/tribe | 0.48 | 0.70 | 0.74 | 0.85 |
|  | Other Backward Classes | 0.47 | 0.70 | 0.68 | 0.93 |
|  | Others | a | a | a | a |
|  |  |  |  |  |  |
| **Telangana** | | 0.55 | 0.74 | 0.71 | 0.82 |
| Residence | |  |  |  |  |
|  | Urban | 0.49 | 0.71 | 0.66 | 0.89 |
|  | Rural | 0.59 | 0.69 | 0.78 | 0.78 |
| Education | |  |  |  |  |
|  | <5 years | 0.79 | 0.64 | 0.88 | 0.75 |
|  | 5-9 years | 0.59 | 0.67 | 0.74 | 0.82 |
|  | 10 years plus | 0.51 | 0.73 | 0.62 | 0.89 |
| Wealth status | |  |  |  |  |
|  | Low | 0.61 | 0.72 | 0.78 | 0.78 |
|  | Middle | 0.56 | 0.68 | 0.76 | 0.78 |
|  | High | 0.49 | 0.72 | 0.63 | 0.93 |
| Caste |  |  |  |  |  |
|  | Scheduled caste/tribe | 0.54 | 0.71 | 0.74 | 0.82 |
|  | Other Backward Classes | 0.55 | 0.69 | 0.73 | 0.82 |
|  | Others | 0.48 | 0.70 | 0.68 | 0.93 |

a = cell count less than 50 cases (unweighted)

[ ] = cell count between 50 -100 cases (unweighted)
