# Supplementary material for: Key drivers of fertility levels and differentials in India, at the national, state and population subgroup levels, 2015–2016: An application of Bongaarts’ proximate determinants model
Source: PLoS One. 2022 Feb 7;17(2):e0263532. doi: 10.1371/journal.pone.0263532 (PMC8820640; doi:10.1371/journal.pone.0263532)
Supplement: S5 Table — (DOCX) [file pone.0263532.s005.docx]

**S5 Table: Estimates of the indices of four key proximate determinants of fertility, actual TFR, estimated TFR and Residual, and estimates of percent contribution of each proximate determinant to fertility reduction: National and by state within TFR groupings, India 2015-16**

|  | **Indices** | | | | **Fertillity** | | | **% contribution of determinant to  fertility reduction from total fecundity§** | | | |
| --- | --- | --- | --- | --- | --- | --- | --- | --- | --- | --- | --- |
| **TFR Groupings**  National/State | **Marriage  (Cm)** | **Contra- ception  (Cc)** | **Induced  Abortion  (Ca)** | **Postpartum  Infecund- ability  (Ci)** | **TFRa*** | **TFRe**** | **Residual  †** | **Marriage** | **Contra- ception** | **Abortion** | **Postpartum Infecund- ability** |
| **India (National)** | 0.59 | 0.70 | 0.71 | 0.78 | 2.18 | 2.48 | 0.30 | 36.0 | 24.1 | 23.4 | 16.4 |
|  |  |  |  |  |  |  |  |  |  |  |  |
| **TFRs of 3.0 - 3.9** |  |  |  |  |  |  |  |  |  |  |  |
| Bihar | 0.72 | 0.86 | 0.79 | 0.73 | 3.41 | 3.90 | 0.49 | 31.7 | 15.0 | 22.3 | 31.0 |
| Meghalaya | 0.54 | 0.83 | 0.72 | 0.75 | 3.04 | 2.69 | -0.35 | 43.4 | 13.2 | 23.3 | 20.1 |
| **TFRs of 2.5 - 2.9** |  |  |  |  |  |  |  |  |  |  |  |
| Jharkhand | 0.65 | 0.78 | 0.73 | 0.73 | 2.55 | 2.90 | 0.36 | 33.1 | 19.1 | 23.7 | 24.1 |
| Manipur | 0.52 | 0.85 | 0.69 | 0.75 | 2.61 | 2.51 | -0.10 | 44.6 | 10.9 | 25.3 | 19.2 |
| Nagaland | 0.48 | 0.82 | 0.65 | 0.93 | 2.74 | 2.61 | -0.13 | 51.7 | 13.5 | 29.7 | 5.1 |
| Uttar Pradesh | 0.59 | 0.72 | 0.69 | 0.82 | 2.74 | 2.64 | -0.10 | 37.0 | 22.7 | 26.0 | 14.3 |
| **TFRs of 2.1 - 2.4** |  |  |  |  |  |  |  |  |  |  |  |
| Arunachal Pradesh | 0.59 | 0.80 | 0.67 | 0.68 | 2.10 | 2.32 | 0.21 | 34.1 | 14.6 | 26.3 | 25.1 |
| Assam | 0.63 | 0.67 | 0.67 | 0.68 | 2.21 | 2.08 | -0.13 | 27.8 | 24.4 | 24.4 | 23.5 |
| Chhattisgarh | 0.55 | 0.71 | 0.66 | 0.82 | 2.23 | 2.31 | 0.08 | 38.3 | 22.2 | 26.5 | 13.1 |
| Madhya Pradesh | 0.60 | 0.73 | 0.68 | 0.78 | 2.32 | 2.55 | 0.23 | 34.7 | 21.8 | 26.8 | 16.7 |
| Mizoram | 0.40 | 0.78 | 0.62 | 0.89 | 2.27 | 1.86 | -0.41 | 52.2 | 14.2 | 27.0 | 6.7 |
| Rajasthan | 0.63 | 0.65 | 0.68 | 0.89 | 2.40 | 2.68 | 0.28 | 33.3 | 31.1 | 27.2 | 8.4 |
| **TFRs 1.8 - 2.0** |  |  |  |  |  |  |  |  |  |  |  |
| Andhra Pradesh | 0.59 | 0.69 | 0.70 | 0.89 | 1.83 | 2.75 | 0.93 | 37.8 | 27.5 | 26.2 | 8.6 |
| Gujarat | 0.57 | 0.74 | 0.66 | 0.89 | 2.03 | 2.71 | 0.68 | 40.0 | 21.8 | 29.7 | 8.5 |
| Haryana | 0.57 | 0.65 | 0.67 | 0.82 | 2.05 | 2.20 | 0.15 | 35.1 | 26.8 | 25.3 | 12.7 |
| Himachal Pradesh | 0.50 | 0.69 | 0.62 | 0.93 | 1.88 | 2.15 | 0.28 | 42.9 | 22.8 | 29.9 | 4.5 |
| Jammu & Kashmir | 0.42 | 0.69 | 0.67 | 0.78 | 2.01 | 1.65 | -0.36 | 46.0 | 20.0 | 21.2 | 12.9 |
| Karnataka | 0.55 | 0.75 | 0.71 | 0.82 | 1.80 | 2.62 | 0.82 | 42.1 | 19.9 | 23.8 | 14.2 |
| Maharashtra | 0.55 | 0.68 | 0.72 | 0.82 | 1.87 | 2.37 | 0.50 | 39.4 | 25.6 | 21.7 | 13.3 |
| Odisha | 0.57 | 0.70 | 0.72 | 0.62 | 2.05 | 1.94 | -0.11 | 32.3 | 20.6 | 19.0 | 28.1 |
| Uttarakhand | 0.51 | 0.70 | 0.67 | 0.82 | 2.07 | 2.13 | 0.06 | 41.2 | 21.7 | 24.7 | 12.4 |
| **TFRs below 1.8** |  |  |  |  |  |  |  |  |  |  |  |
| Goa | 0.45 | 0.82 | 0.72 | 0.73 | 1.66 | 2.09 | 0.43 | 48.9 | 11.8 | 20.1 | 19.3 |
| Kerala | 0.49 | 0.74 | 0.67 | 0.85 | 1.56 | 2.28 | 0.72 | 45.5 | 18.9 | 25.4 | 10.3 |
| Punjab | 0.44 | 0.57 | 0.59 | 0.89 | 1.62 | 1.44 | -0.18 | 40.3 | 28.0 | 25.9 | 5.8 |
| Sikkim | 0.44 | 0.73 | 0.53 | 0.68 | 1.17 | 1.24 | 0.06 | 38.1 | 14.6 | 29.5 | 17.8 |
| Tamil Nadu | 0.50 | 0.73 | 0.69 | 0.93 | 1.70 | 2.59 | 0.89 | 48.2 | 21.5 | 25.3 | 5.0 |
| Telangana | 0.55 | 0.74 | 0.71 | 0.82 | 1.78 | 2.58 | 0.81 | 41.6 | 20.5 | 23.8 | 14.1 |
| Tripura | 0.63 | 0.64 | 0.64 | 0.62 | 1.68 | 1.74 | 0.06 | 24.9 | 24.1 | 24.6 | 26.4 |
| West Bengal | 0.67 | 0.59 | 0.69 | 0.62 | 1.77 | 1.83 | 0.06 | 22.8 | 29.2 | 20.8 | 27.2 |

* = Actual Total Fertility Rates

** = Estimate Total Fertility Rates

† Residual = The difference between TFRe and TFRa.

§ The proportionate reduction in fertility (from the Total Fecundity Rate to the actual Total Fertility Rate) that is attributable to each proximate determinant
